# Hypomyelinating Leukodystrophy 10 (HLD10)-Associated Mutations of PYCR2 Form Large Size Mitochondria, Inhibiting Oligodendroglial Cell Morphological Differentiation

**DOI:** 10.3390/neurolint14040085

**Published:** 2022-12-16

**Authors:** Tomohiro Torii, Remina Shirai, Risa Kiminami, Satoshi Nishino, Takanari Sato, Sui Sawaguchi, Nana Fukushima, Yoichi Seki, Yuki Miyamoto, Junji Yamauchi

**Affiliations:** 1 Laboratory of Ion Channel Pathophysiology, Graduate School of Brain Science, Doshisha University, Kyotanabe 610-0394, Japan; 2 Laboratory of Molecular Neurology, Tokyo University of Pharmacy and Life Sciences, Hachioji 192-0392, Japan; 3 Department of Pharmacology, National Research Institute for Child Health and Development, Tokyo 157-8535, Japan; 4 Diabetic Neuropathy Project, Tokyo Metropolitan Institute of Medical Science, Tokyo 156-8506, Japan

**Keywords:** HLD10, PYCR2, oligodendrocyte, differentiation, mitochondrion

## Abstract

Hypomyelinating leukodystrophy 10 (HLD10) is an autosomal recessive disease related to myelin sheaths in the central nervous system (CNS). In the CNS, myelin sheaths are derived from differentiated plasma membranes of oligodendrocytes (oligodendroglial cells) and surround neuronal axons to achieve neuronal functions. Nucleotide mutations of the pyrroline-5-carboxylate reductase 2 (PYCR2) gene are associated with HLD10, likely due to PYCR2’s loss-of-function. PYCR2 is a mitochondrial residential protein and catalyzes pyrroline-5-carboxylate to an amino acid proline. Here, we describe how each of the HLD10-associated missense mutations, Arg119-to-Cys [R119C] and Arg251-to-Cys [R251C], lead to forming large size mitochondria in the FBD-102b cell line, which is used as an oligodendroglial cell differentiation model. In contrast, the wild type proteins did not participate in the formation of large size mitochondria. Expression of each of the mutated R119C and R251C proteins in cells increased the fusion abilities in mitochondria and decreased their fission abilities relatively. The respective mutant proteins, but not wild type proteins also decreased the activities of mitochondria. While cells expressing the wild type proteins exhibited differentiated phenotypes with widespread membranes and increased expression levels of differentiation marker proteins following the induction of differentiation, cells harboring each of the mutant proteins did not. Taken together, these results indicate that an HLD10-associated PYCR2 mutation leads to the formation of large mitochondria with decreased activities, inhibiting oligodendroglial cell morphological differentiation. These results may reveal some of the pathological mechanisms in oligodendroglial cells underlying HLD10 at the molecular and cellular levels.

## 1. Introduction

Pelizaeus–Merzbacher disease (PMD) is classified as a hypomyelinating and/or dysmyelinating hereditary disorder of the central nervous system (CNS) and is primarily linked to oligodendrocytes (also called oligodendroglial cells) [1,2,3,4]. It is thought that normal myelination never occurs in PMD. PMD is now known as the prototypic disorder of hypomyelinating leukodystrophies (HLDs) [1,2,3,4]. These diseases are rare, affecting one out of every 250,000 to 500,000 people [1,2,3,4]. The gene responsible for HLD1 encodes proteolipid protein 1 (PLP1), which is the major myelin structural protein [1,2,3,4]. Myelin sheaths, which are generated by oligodendroglial cells, contribute to both the propagation of saltatory conduction and to protecting neuronal axons from stresses such as physical and physiological stresses [5,6,7,8]. Thus, they play a key role in CNS functions [5,6,7,8].

Due to a rapid advance in DNA analysis technologies, including next-generation sequencing (NGS), there has been increasing identification of many expected (related directly to myelin sheath-generating molecules) and unexpected (expected to be indirectly related to myelin sheath-generating molecules) disease-responsible genes. The gene responsible for HLD10 (OMIM No. 616420) is one such unexpected gene [9,10,11,12,13,14]. It encodes the pyrroline-5-carboxylate reductase 2 (pycr2) gene. PYCR2 catalyzes pyrroline-5-carboxylate to an amino acid proline. This reaction is the last catabolic step of proline biosynthesis [15,16]. HLD10 is associated with some PYCR2 mutations and is thought to be loss-of-function for the enzymatic activities [9,10,11,12,13,14]. Brain imaging demonstrates that HLD10 displays severe hypomyelination phenotypes with thin myelin sheaths in the corpus callosum and several other brain regions [9,10,11,12,13,14,15,16]. To date, there is no known specific therapeutic strategy or drug for HLD10.

Here we describe how each of the originally identified HLD10-associated mutations (Arg119-to-Cys [R119C] and Arg251-to-Cys [R251C]) [9] lead to forming large size mitochondria in the FBD-102b cell line, which is the oligodendroglial cell model [17,18,19]. Expression of the respective mutant proteins (R119C and R251C) in cells exhibited increased fusion abilities in mitochondria and decreased their fission abilities. In contrast, cells expressing the wild type PYCR2 proteins did not have large size mitochondria. Following the induction of differentiation, cells harboring wild types displayed differentiated phenotypes with widespread membranes and increased differentiation and myelin marker protein expression, whereas cells harboring the mutants did not. These results could enhance our understanding of the potential molecular and cellular pathological mechanisms underlying HLD10.

## 2. Material and Methods

### 2.1. Primary and Secondary Antibodies and Chemicals

Primary antibodies were purchased as described below. Mouse monoclonal anti-myelin basic protein specific for myelin-forming glial cells (MBP, Cat. No. 836506; immunoblotting [IB], 1/500) from BioLegend (San Diego, CA, USA); mouse monoclonal anti-2′,3′-cyclic nucleotide 3′-phospho-diesterase specific for myelin-forming glial cells (CNPase, Cat. No. 5664; IB, 1/500) from Cell Signaling Technology (Danvers, MA, USA); mouse monoclonal alpha 1 sodium potassium ATPase (ATP1A1; Cat. No. sc-21712; IB, 1/100) as the general control membrane protein marker, mouse monoclonal oligodendroglial cell-rich ErbB4 (Cat. No. sc-8050; IB, 1/100), mouse monoclonal pan-ErbB receptors (Cat. No. sc-101; IB, 1/100) as the membrane protein, mouse monoclonal anti-myelin-forming glial cell lineage-specific Sox10 (Cat. No. sc-365692; IB, 1/500), and mouse monoclonal anti-lysosomal-associated membrane protein 1 (LAMP1, Cat. No. sc-20011; immunofluorescence [IF], 1/200) from Santa Cruz Biotechnology (Santa Cruz, CA, USA); mouse monoclonal anti-actin (control actin, Cat. No. M177-3; IB, 1/40,000) and mouse monoclonal anti-endoplasmic reticulum (ER)-resident Lys-Asp-Glu-Leu oligopeptide antigen (KDEL, Cat. No. M181-3; IF, 1/500) from MBL (Aichi, Japan); mouse monoclonal anti-Golgi matrix protein of 130 kDa (GM130, Cat. No. 610823; IF, 1/500) and mouse monoclonal anti-mitochondrion-specific mitochondrial heat shock protein family D member 1 (HSPD1, Cat. No. 611562; IF, 1/5000) from BD Biosciences (Franklin Lakes, NJ, USA).

The following secondary antibodies were used in immunoblotting experiments: anti-mouse or rabbit IgG F(ab’) conjugated with horseradish peroxidase (Cat. Nos. 458 or 330; IB, 1/5000) was purchased from MBL. The following secondary antibodies for immunofluorescence experiments were purchased: anti-mouse or rabbit IgG (H + L) conjugated with Alexa Fluor 594 (Cat. Nos. A-11005 or A-11012; IF, 1/500) was purchased from Thermo Fisher Scientific (Waltham, MA, USA).

Mitochondrial JC-1 dye was purchased from Dojindo Laboratories (Kumamoto, Japan) and used according to the manufacturer’s instructions.

### 2.2. Construction of Plasmids

The full-length coding region of the human pycr2 gene (Gene ID: 29920) was amplified from the human brain cDNA library (Fujifilm, Tokyo, Japan) using Gflex DNA polymerase (Takara Bio, Shiga, Japan) and ligated into pEGFP-N3 with a Mighty mix DNA ligation kit (Takara Bio) according to the manufacturer’s instructions. The R119C and R251C mutations (OMIM ID: 616420) were generated using a PrimeSTAR mutagenesis basal kit (Takara Bio) according to the manufacturer’s instructions. DNA sequences were confirmed by Fasmac sequencing service (Kanagawa, Japan).

### 2.3. Cell Culture and Differentiation

The Africa green monkey COS-7 cell line, which exhibits a wide flat cell phenotype suitable for determining the cellular localization of gene products, was purchased from the JCRB Cell Bank (Osaka, Japan). Cells were cultured on cell culture dishes (Greiner, Oberösterreich, Germany) in a culture medium consisting of Dulbecco’s modified eagle medium (DMEM) (Nacalai Tesque, Kyoto, Japan) containing 10% heat-inactivated fetal bovine serum (FBS, Thermo Fisher Scientific) and PenStrep (Thermo Fisher Scientific) in 5% CO_2_ at 37 °C. The oligodendroglial FBD-102b cell line (a mouse brain oligodendroglial precursor cell line) was kindly provided by Dr. Y. Tomo-oka (Tokyo University of Science, Chiba, Japan and Riken, Saitama, Japan). FBD-102b cells were cultured on cell culture dishes in a culture medium consisting of a DMEM/nutrient mixture F-12 (Nacalai Tesque, Kyoto, Japan) containing 10% heat-inactivated FBS and PenStrep in 5% CO_2_ at 37 °C. These cells, in an environment with insufficient CO_2_ in a microscopic system, were incubated in Leibovitz’s L-15 medium containing 10% heat-inactivated FBS and PenStrep at 37 °C.

To induce differentiation, FBD-102b cells were cultured for 5 days in the same culture medium without FBS on cell culture dishes coated with poly-L-lysin (Nacalai Tesque) in 5% CO_2_ at 37 °C. Cells with widespread membranes (more than 75 mm square fields; Image J software [Bethesda, MD, USA]) were identified as differentiated. Along with these observations, the expression levels of myelin marker proteins were confirmed by immunoblotting techniques. FBD-102b cells form widespread membranes and express myelin marker proteins [17,18,19,20].

We confirmed that COS-7 cells and FBD-102b cells were viable under each experimental condition by verifying that attached trypan-blue (Nacalai Tesque)-incorporating cells made up less than 5% of all cells in each culture.

### 2.4. Transfection

Cells were transfected with the respective plasmids using a ScreenFect A or ScreenFect A Plus transfection kit (Fujifilm) according to the manufacturer’s instructions. The medium was replaced 4 h after transfection. Transfected cells were generally used for experiments 48 h after transfection in cell biological and biochemical experiments. To collect FBD-102b cells stably harboring the respective constructs, we transfected cells with plasmids encoding the respective DNAs in a 3.5 cm cell culture dish. Growth medium containing gradually increasing concentrations of G418 (500 to 2000 mg/mL, Nacalai Tesque) was changed every 2 to 3 days. After more than 14 days, G418-resistant clones were mixedly collected and further cultured for an additional 14 days so that their phenotypes could be compared to those of other stable clones.

We confirmed that COS-7 cells and FBD-102b cells were viable under each experimental condition by verifying that attached trypan-blue (Nacalai Tesque)-incorporating cells made up less than 5% of all cells in each culture.

### 2.5. Capturing Confocal Images

For capturing still images, coverslips loaded with cells fixed with 4% paraformaldehyde or 100% cold methanol were blocked with Blocking One (Nacalai Tesque). These were then incubated with primary antibodies followed by secondary antibodies conjugated with Alexa Fluor dyes according to the manufacturer’s instructions. The coverslips on each slide glass were mounted with Vectashield with 4′,6-diamidino-2-phenylindole (DAPI) kit (Vector Laboratories, Burlingame, CA, USA). Tag image file format (TIFF) images were collected through a microscope equipped with a laser-scanning Fluoview apparatus (FV1000D/FV1200/FV3000, Olympus, Tokyo, Japan). For capturing live images, an image was taken once every 4 s through a microscope equipped with a laser scanning device adjusted with a 3-fold intensity laser. These images were processed using Fluoview software (Olympus). The resulting color images were analyzed in Image J software. Each image in each figure is representative of at least 3 independent experimental results.

### 2.6. Polyacrylamide Gel Electrophoresis and Immunoblotting

Cells were lysed in lysis buffer A (50 mM HEPES-NaOH, pH 7.5, 150 mM NaCl, 20 mM MgCl_2_, 1 mM phenylmethane sulfonylfluoride, 1 μg/mL leupeptin, 1 mM EDTA, 1 mM Na_3_VO_4_, 10 mM NaF, and 0.5% NP-40). After centrifugation, the supernatants were mixed with sample buffer (Nacalai Tesque). Then, the samples were separated on polyacrylamide gels (Nacalai Tesque). The electrophoretically separated proteins were transferred onto polyvinylidene difluoride membranes (Merck-Millipore, Darmstadt, Germany) and blocked with Blocking One, then immunoblotted with primary antibodies followed by secondary antibodies conjugated with horseradish peroxidase. The bound antibodies were incubated with ImmunoStar Zeta (Fujifilm) and detected by X-ray film (Fujifilm) exposure. Images were captured as TIFF files using LiDE scanners (Canon, Tokyo, Japan) and processed using the accompanying LiDE driver software (Canon). The band pixels were measured in Image J software. Each image in each figure is representative of at least 3 independent experimental results.

### 2.7. Statistical Analysis

Values are means ± standard deviation (SD) from separate experiments. Intergroup comparisons were made using the unpaired Student’s *t*-test using Excel (Microsoft, Redmond, WA, USA). A one-way analysis of variance (ANOVA) was followed by a Tukey’s multiple comparison test using Graph Pad Prism (GraphPad Software, San Diego, CA, USA). Differences were considered statistically significant when *p* < 0.05.

### 2.8. Ethics Statement

Gene recombination techniques were performed in accordance with a protocol approved by the Tokyo University of Pharmacy and Life Sciences Gene and Animal Care Committee (Approval Nos. LS28-20 and LSR3-011).

## 3. Results

### 3.1. R119C or R251C Mutant Proteins Are Specifically Localized in Mitochondria and Involved in Forming Large Size Mitochondria

**Figure 1:**
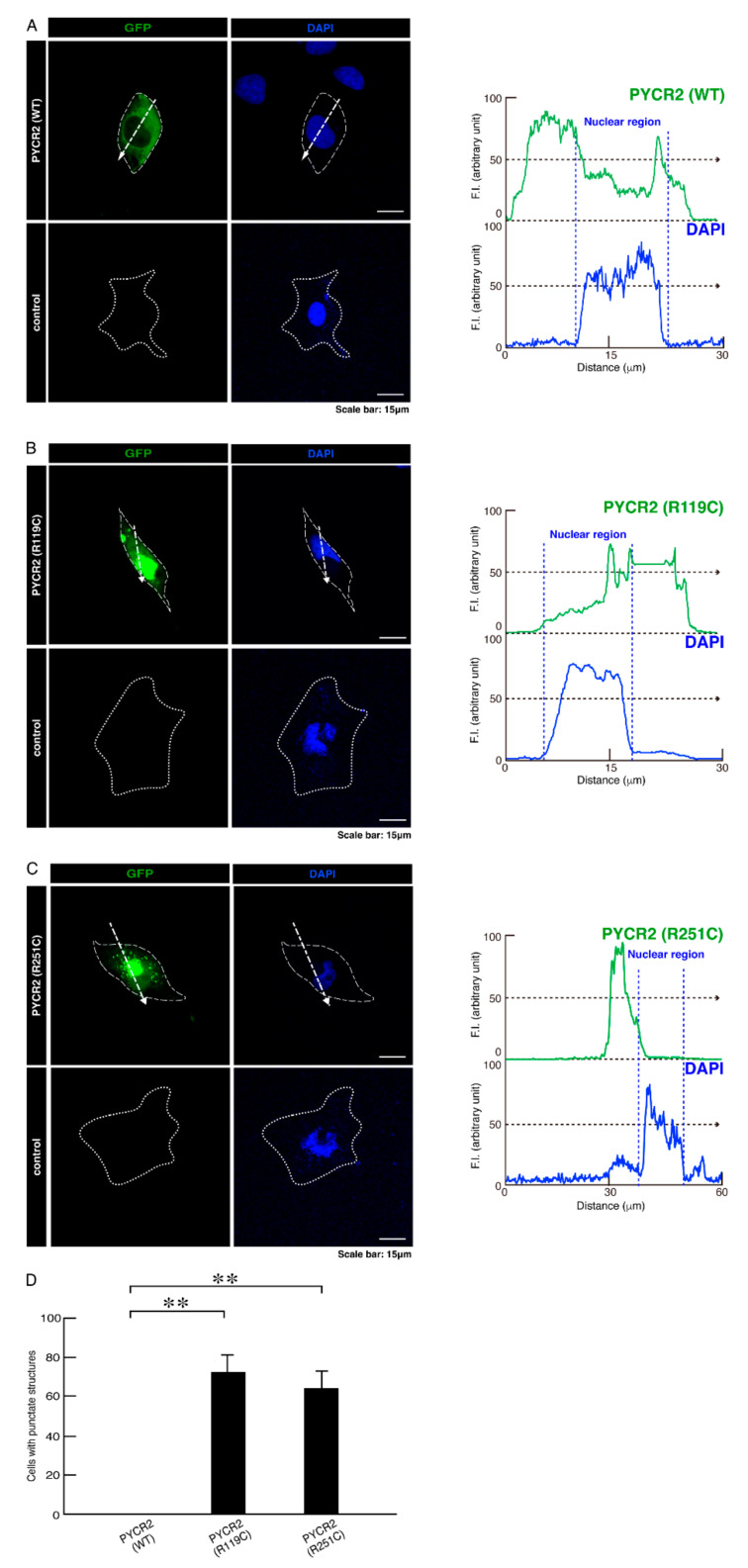
The PYCR2 R119C and R251C mutated proteins, but not the wild type PYCR2 proteins, exhibit structures like large size aggregates or organelles in cells. (**A**–**C**) COS-7 cells were transfected with the plasmid encoding the GFP-tagged wild type (WT) PYCR2 or PYCR2 with the R119C or R251C mutation. Transfected cells were detected with PYCR2 proteins (green) and nuclear DAPI (blue). Control images are also shown. The approximate outline of the cell is surrounded by white dotted lines. Scan plots were performed along the white dotted lines in the direction of the arrows in the color images. Graphs showing the fluorescence intensities (F.I., arbitrary units) along the lines in the direction of the arrows were depicted in the bottom panels. (**D**) Percentages of cells with large size aggregate- or organelle-like structures were statistically assessed (**, *p* < 0.01; *n* = 10 fields).

In order to investigate whether HLD10-associated R119C or R251C mutant proteins of PYCR2 are distributed in punctate organelles such as mitochondria throughout cytoplasmic regions, we transfected the respective plasmids encoding GFP-tagged wild type, R119C, or R251C mutant proteins into COS-7 cells, which are wide flat cells suitable for studies using transfection and in turn identifying the cellular localization of the purposed proteins [21,22]. While wild type PYCR2 proteins were distributed throughout cytoplasmic regions, R119C or R251C mutant proteins were present as intracellular structures like large size aggregates or organelles outside the nucleus (Figure 1A–D and Figures S1–S3).

Next, we sought to determine whether wild type, R119C, or R251C proteins are localized in other organelles. First, we stained cells expressing wild type, R119C, or R251C proteins of PYCR2 with an antibody against proteins with KDEL at their C-terminus, which is the specific ER antigen. Neither R119C, R251C, nor wild type proteins exhibited major co-localization with ER antigens (Figures S4–S6). Then, we stained cells expressing wild type, R119C, or R251C proteins of PYCR2 with an antibody against GM130, which is the specific Golgi body antigen. Neither R119C, R251C, nor wild type proteins exhibited major co-localization with GM130 antigens (Figure S7–S9). We also stained cells expressing wild type, R119C, or R251C proteins of PYCR2 with an antibody against LAMP1, which is the specific lysosome antigen. Neither R119C, R251C, nor wild type proteins exhibited major co-localization with LAMP1 antigens (Figure S10–S12).

**Figure 2:**
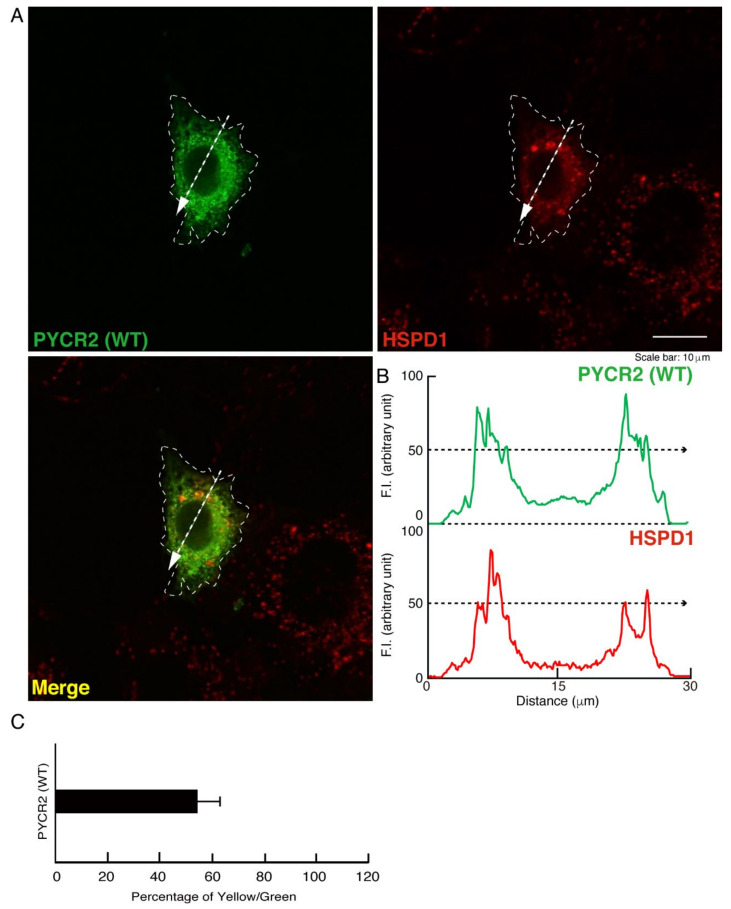
The wild type proteins are partially co-localized with the mitochondria. (**A**,**B**) COS-7 cells were transfected with the plasmid encoding the GFP-tagged wild type (WT) PYCR2 and stained with an antibody against the mitochondrion-specific antigen HSPD1 (red). GFP-tagged WT PYCR2 proteins were identified by their green fluorescence color. The approximate outline of the cell is surrounded by white dotted lines. Scan plots were performed along the white dotted lines in the direction of the arrows in the color images (green and red as well as merged images). Graphs showing the fluorescence intensities (F.I., arbitrary units) along the lines in the direction of the arrows were depicted in the right bottom panels. (**C**) Percentage of merged yellow fluorescence pixel values per green fluorescence ones are shown in the graph.

**Figure 3:**
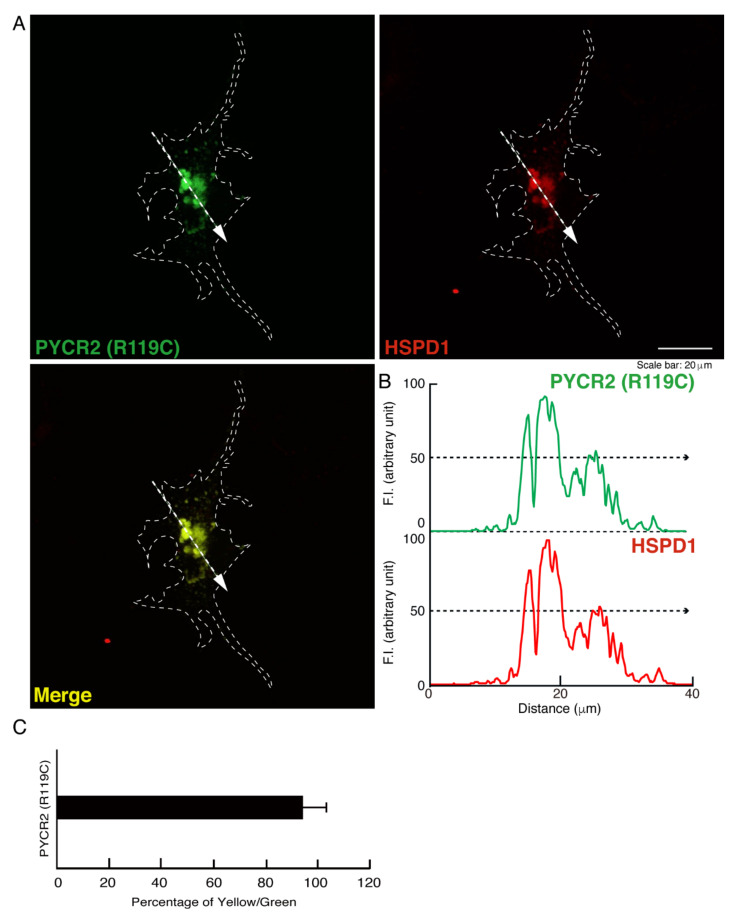
The R119C proteins are significantly co-localized with the large size mitochondria. (**A**,**B**) COS-7 cells were transfected with the plasmid encoding the GFP-tagged PYCR2 R119C and stained with an antibody against the mitochondrion-specific antigen HSPD1 (red). GFP-tagged PYCR2 R119C proteins were identified by their green fluorescence color. The approximate outline of the cell is surrounded by white dotted lines. Scan plots were performed along the white dotted lines in the direction of the arrows in the color images (green and red as well as merged images). Graphs showing the fluorescence intensities (F.I., arbitrary units) along the lines in the direction of the arrows were depicted in the right bottom panels. (**C**) Percentage of merged yellow fluorescence pixel values per green fluorescence ones are shown in the graph.

**Figure 4:**
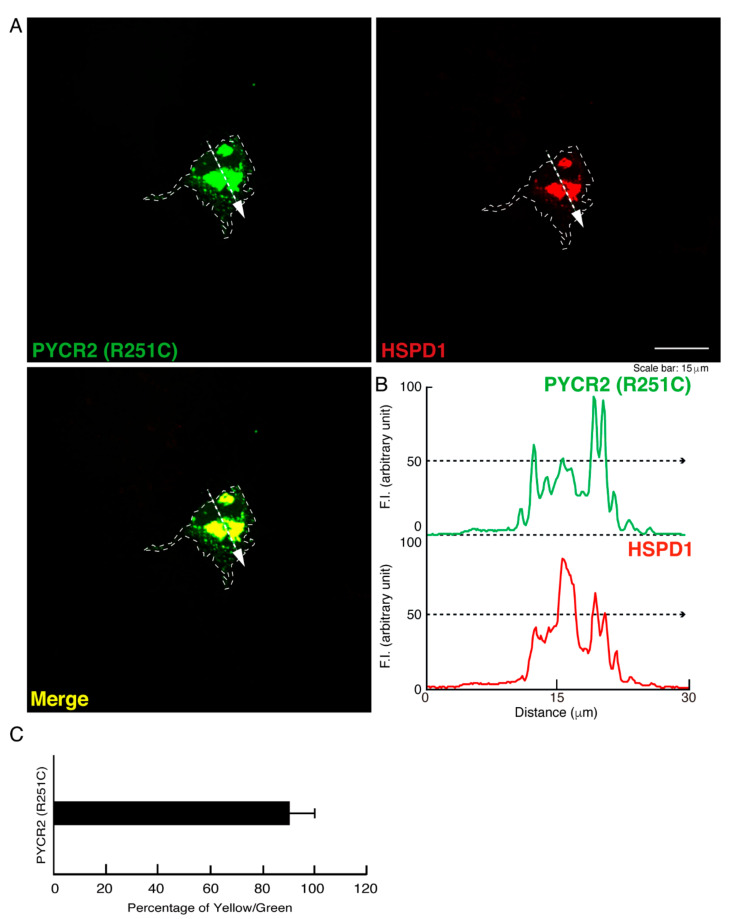
The R251C proteins are significantly co-localized with the large size mitochondria. (**A**,**B**) COS-7 cells were transfected with the plasmid encoding the GFP-tagged PYCR2 R251C and stained with an antibody against the mitochondrion-specific antigen HSPD1 (red). GFP-tagged PYCR2 R251C proteins were identified by their green fluorescence color. The approximate outline of the cell is surrounded by white dotted lines. Scan plots were performed along the white dotted lines in the direction of the arrows in the color images (green and red as well as merged images). Graphs showing the fluorescence intensities (F.I., arbitrary units) along the lines in the direction of the arrows were depicted in the right bottom panels. (**C**) Percentage of merged yellow fluorescence pixel values per green fluorescence ones are shown in the graph.

We tried to examine whether intracellular structures like large size organelles correspond to mitochondria. We stained cells expressing wild type, R119C, or R251C proteins of PYCR2 with an antibody against mitochondrion-specific antigen HSPD1, which is a mitochondrion-specific chaperone protein, as the specific antigens for mitochondria. While wild type PYCR2 proteins were partially stained with an anti-HSPD1 antibody, large size organelles observed in cells expressing R119C or R251C proteins were greatly stained with an antibody against HSPD1 (Figure 2A–C, Figure 3A–C and Figure 4A–C). Similar results (Figure S13–S15) were observed in studies using FBD-102b cells, which are cell models undergoing oligodendroglial cell morphological differentiation [17,18,19,20].

Taken together with the results described above, these results suggest that mitochondria with HLD10-associated R119C or R251C mutant proteins result in forming large size structures.

### 3.2. R119C or R251C Mutant Proteins Comparatively Increase Mitochondrial Fusion Activities and Decrease Membrane Potential Activities

**Figure 5:**
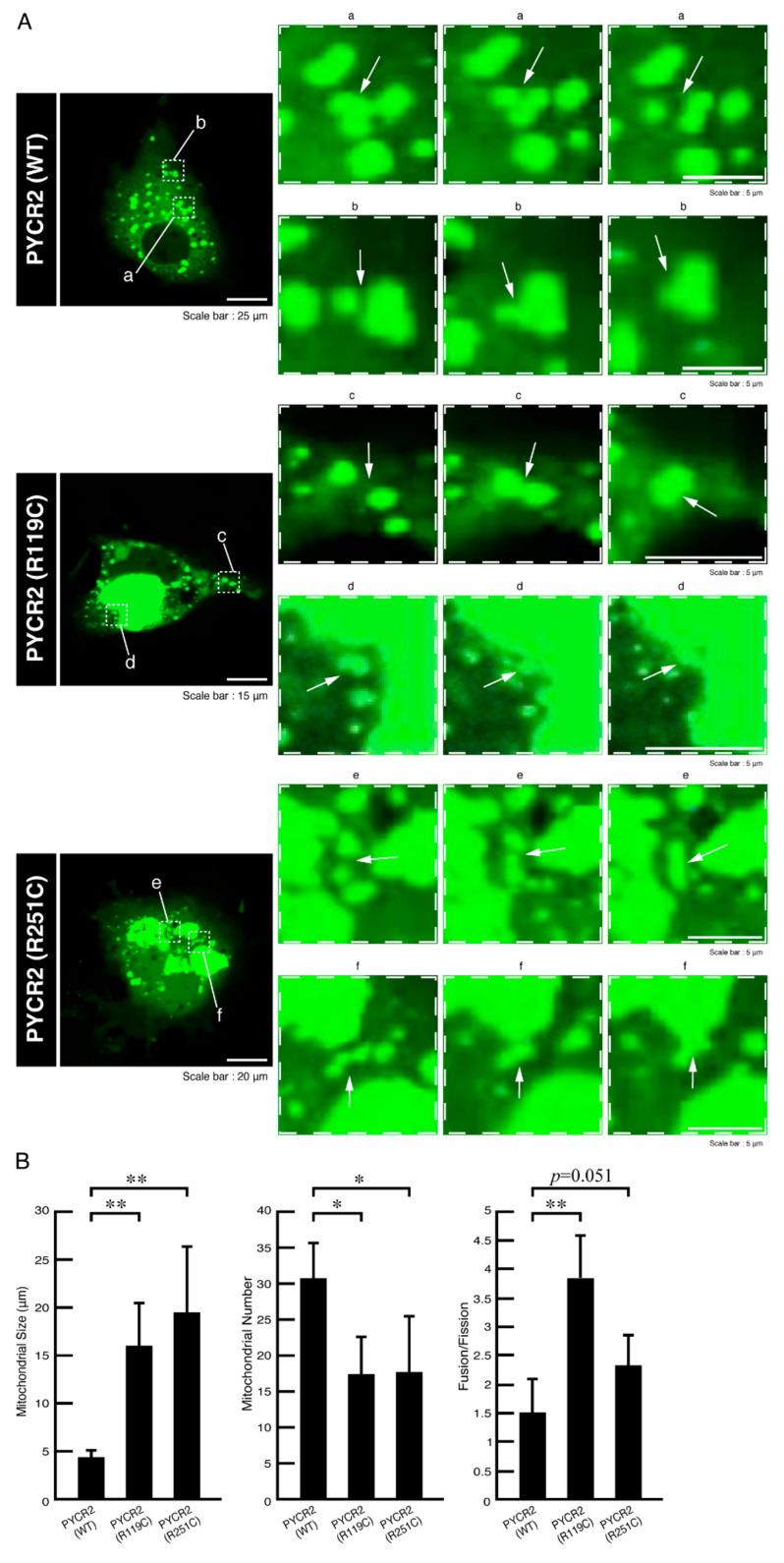
The R119C or R251C proteins preferentially increase mitochondrial fusion whereas the wild type ones exhibit normal size mitochondoria. (**A**) COS-7 cells were transfected with the plasmid encoding the respective GFP-tagged PYCR2 constructs. An image of the respective transfected cells was taken once every 4 s for 1 h. GFP-tagged proteins, which were taken with a 3-fold strength laser through a microscope equipped with a laser-scanning Fluoview apparatus, were identified by their green fluorescence color. In the wild type (WT) PYCR2 image, right upper and lower panels were time-lapse magnified images of white dotted squares a and b of the left panel. Mitochondria in dotted square a exhibit fission. Mitochondria in dotted square b exhibit fusion (indicated by the arrows). In the R119C PYCR2 image, right upper and lower panels were time-lapse magnified images of white dotted squares c and d of the left panel. Mitochondria in dotted squares c and d exhibit fusion (indicated by the arrows). In the R251C PYCR2 image, right upper and lower panels were time-lapse magnified images of white dotted squares e and f of the left panel. Mitochondria in dotted squares e and f exhibit fusion (indicated by the arrows). (**B**) The largest mitochondrial sizes per cell were depicted in the left graph (**, *p* < 0.01; *n* = 10 cells). The numbers of mitochondria per cell were depicted in the middle graph (*, *p* < 0.05; *n* = 10 cells). The ratios of fusion per fission of mitochondria were depicted in the right graph (**, *p* < 0.01; *n* = 10 cells).

The following two simple reasons may explain the formation of large size mitochondria: (1) mitochondrial fusion occurs more frequently than fission, and (2) mitochondrial fission rarely occurs [23,24,25]. In order to prove these hypotheses, we recorded time-lapse images in cells expressing wild type, R119C, or R251C proteins of PYCR2 and measured the fusion and fission ratios with various mitochondrial indexes. Mitochondria were indeed large in size and few in number in cells expressing R119C or R251C proteins compared to cells expressing wild type ones (Figure 5A,B). The ratios of fusion and fission were increased compared to cells expressing wild type proteins, suggesting that mitochondrial fusion in cells expressing mutated proteins occurs more frequently than fission (Figure S16).

**Figure 6:**
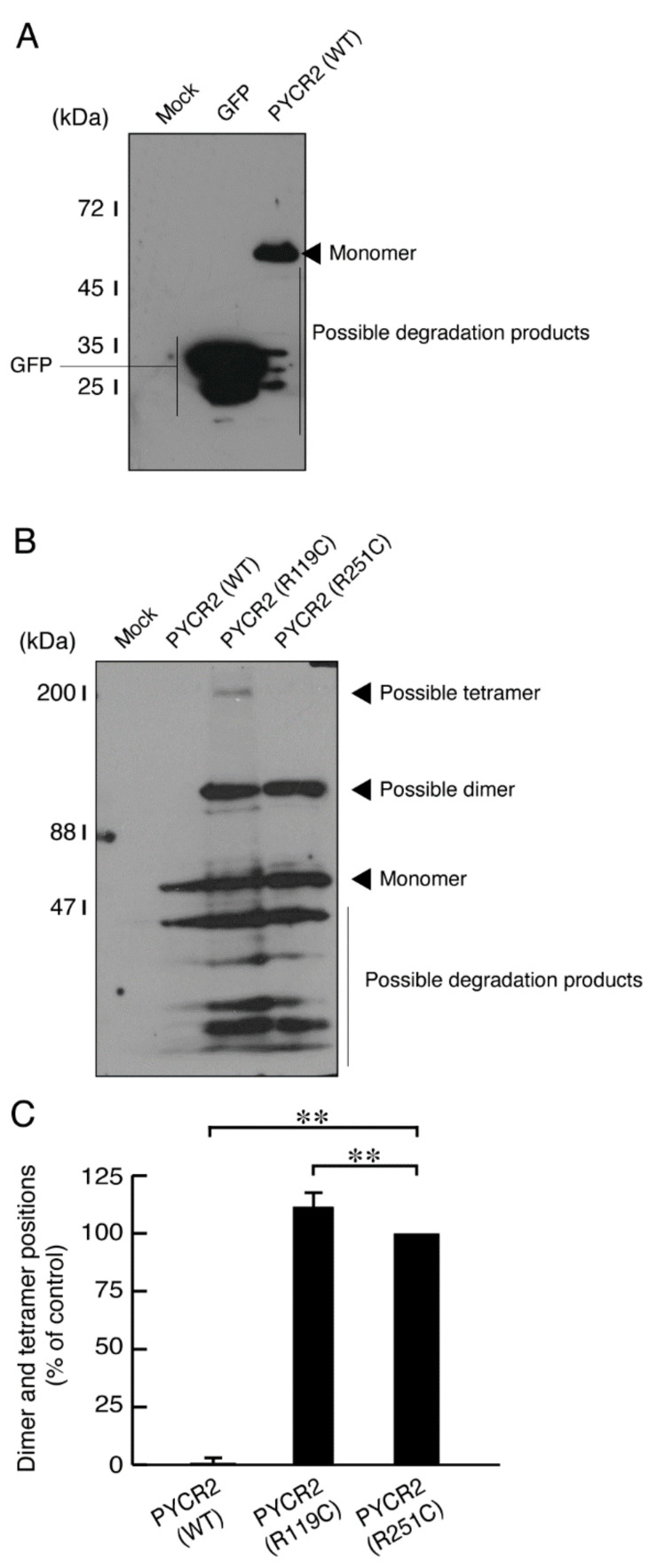
The R119C or R251C proteins, but not the wild type proteins, exhibit dimeric or tetrameric structures in a polyacrylamide gel with a full-size gel image. (**A**) As the control experiments, the lysates of COS-7 cells transfected with mock, an empty vector expressing GFP, or with a plasmid encoding the wild type (WT) were applied to polyacrylamide gel electrophoresis and detected using immunoblotting with an anti-GFP antibody. (**B**) The lysates of COS-7 cells transfected with an empty vector or with a plasmid encoding the wild type (WT) or the respective mutated constructs were applied to polyacrylamide gel electrophoresis and detected using immunoblotting with an anti-GFP antibody. The positions corresponding to monomeric or polymeric structures are shown. (**C**) Immunoreactive bands corresponding to monomeric or polymeric structures were compared and statistically depicted (**, *p* < 0.01; *n* = 3 blots).

Since the fusion ratios were increased in cells expressing R119C or R251C proteins, we asked whether R119C or R251C proteins have a tendency to dimerize or oligomerize for mitochondrial fusion in cells. We applied the lysates of cells expressing wild type, R119C, or R251C proteins to a polyacrylamide gel to separate sizes according to molecular mass. In comparison with the control experiments, the molecular mass of wild type proteins corresponded to monomeric position whereas that of mutated proteins corresponded to dimeric and/or trimeric positions (Figure 6A–C). Although the specific localization of PYCR2 proteins in mitochondrial outer and/or inner membranes has not been identified to date, these results suggest that dimeric and/or trimeric properties of the mutated proteins can contribute to fusion between mitochondria.

**Figure 7:**
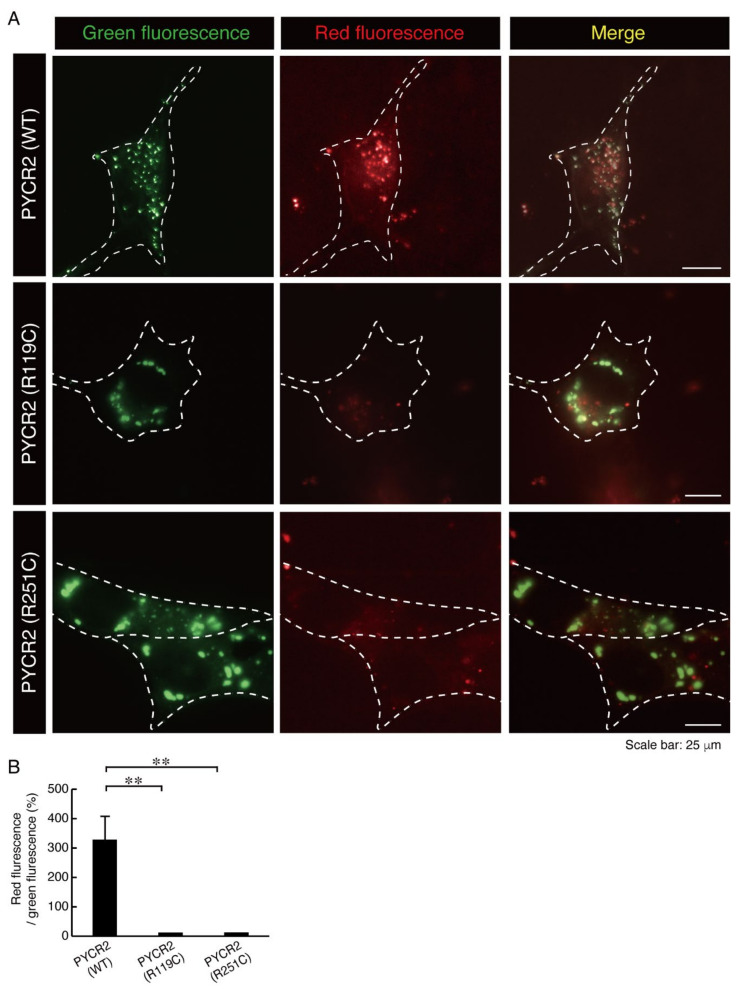
The mutated PYCR2 proteins decrease mitochondrial membrane potentials. (**A**,**B**) FBD-102b cells expressing wild type (WT), R119C, or R251C of PYCR2 (green) were treated with JC-1 dye (red). The approximate outline of the cell is surrounded by dotted white lines. JC-1 was taken up by wild type PYCR2-expressing mitochondria that maintained normal membrane potential. Pixel ratios of red fluorescence per green fluorescence were depicted to be statistically significant (**, *p* < 0.01; *n* = 10 cells).

To clarify whether mutated proteins affect mitochondrial activities such as the membrane potentials, we treated the respective transfected cells with JC-1 dye. When membrane potentials are activated and higher, the JC-1 dye displays red fluorescence where it has accumulated as polymers within the mitochondria [26]. At lower membrane potentials, the JC-1 dye is present as monomers, displaying green fluorescence. Cells expressing wild type proteins exhibited red fluorescence. In contrast, cells expressing the respective mutated proteins failed to exhibit significantly red fluorescence (Figure 7A,B), suggesting that mutated proteins have effects on decreasing mitochondrial activities.

### 3.3. R119C or R251C Mutant Proteins Inhibit Morphological Differentiation with Widespread Membranes

**Figure 8:**
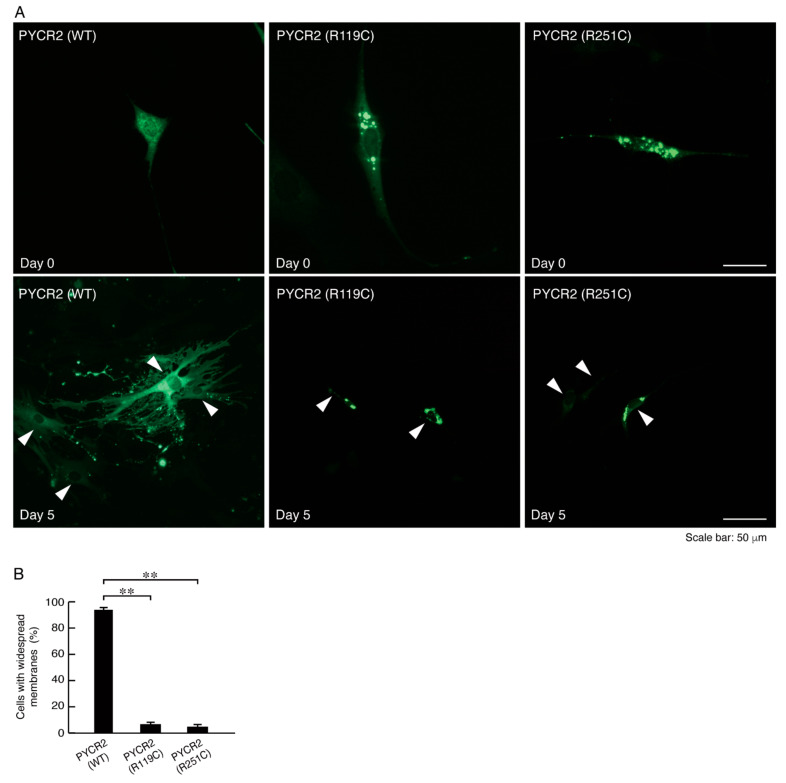
Cells expressing the R119C or R251C proteins, but not the wild type proteins, fail to exhibit oligodindroglial cell morphological differentiation. (**A**,**B**) FBD-102b cells expressing the R119C, R251C proteins, or the wild type proteins were allowed to differentiate for 0 or 5 days. Arrowheads indicate typical differentiated cells with widespread membranes in cells expressing the wild type proteins and undifferentiated cells in cells expressing the R119C or R251C proteins. Differentiated cells were statistically assessed (**, *p* < 0.01; *n* = 10 fields).

**Figure 9:**
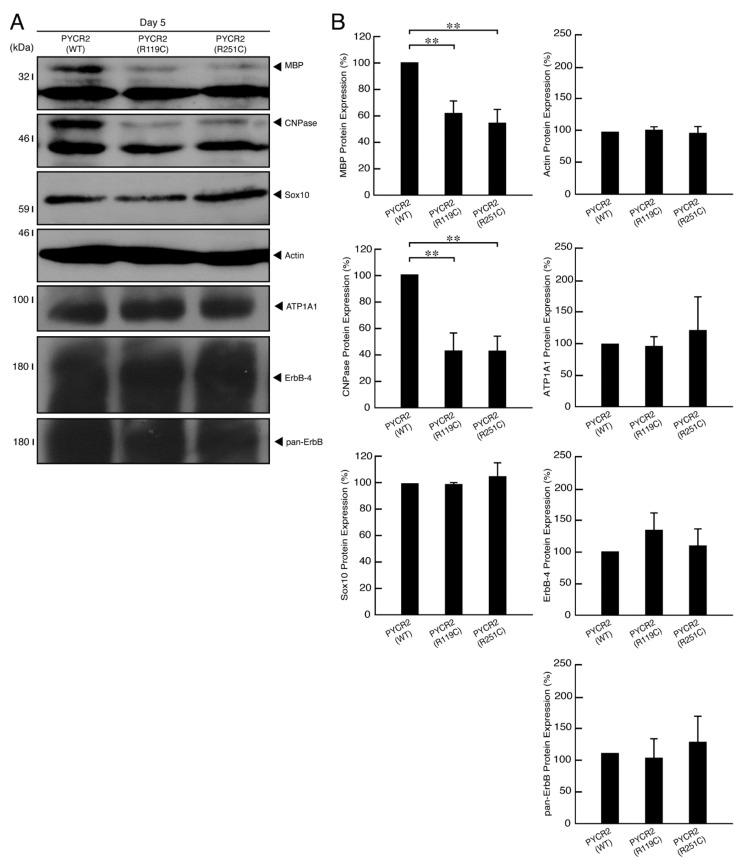
Cells expressing the R119C or R251C proteins, but not the wild type proteins, exhibit decreased expression levels of oligodendroglial cell differentiation marker proteins. (**A**,**B**) The lysates of the respective FBD-102b cells were immunoblotted with an antibody against oligodendroglial cell differentiation marker protein MBP or CNPase, oligodendroglial cell lineage marker Sox10. Additionally, antibodies against actin, ATP1A1, ErbB4, and pan-ErbB were utilized as the control protein markers. Their expression levels are shown statistically compared to their respective controls. (**, *p* < 0.01; *n* = 3 blots for MBP, CNPase, and actin, and *n* = 4 blots for ATP1A1, ErbB4, and pan-ErbB).

To examine whether expression of PYCR2 R119C or R251C proteins in cells results in inhibition of oligodendroglial cell morphological differentiation, we generated cells expressing wild type, R119C, or R251C proteins. Cells expressing wild type PYCR2 proteins exhibited differentiated phenotypes with widespread membranes following the induction of differentiation, as seen in oligodendroglial cells (Figure 8A,B and Figure S17). Cells expressing the respective mutated proteins failed to undergo morphological differentiation, suggesting that defective morphological differentiation by the respective mutated proteins probably leads to hypomyelination phenotypes. These cellular phenotypes were supported with decreased expression levels of marker proteins MBP and CNPase in cells expressing the respective mutated proteins compared to cells expressing the wild type ones (Figure 9A,B). Expression levels of Sox10 proteins as the oligodendrocyte lineage marker and actin proteins as the internal control marker were comparable in the lysates of all types of cells. Taken together with the results of cell morphologies and marker protein expression levels, these results suggest that PYCR2 R119C or R251C proteins inhibit oligodendroglial cell morphological differentiation.

## 4. Discussion

Brain imaging shows that HLD10 is associated with thin myelin sheaths in the corpus callosum and other brain regions [9,10,11,12,13,14,15,16]. These symptoms were supported by our finding that cells harboring the wild type PYCR2 constructs exhibited differentiated phenotypes whereas cells harboring HLD10-associated PYCR2 mutants inhibited morphological differentiation. Since HLD10 displays progressive microcephaly and reduced cerebral white matter volume, HLD10-associated PYCR2 mutations appear to lead to developing abnormalities in the whole brain. PYCR family proteins are composed of PYCR1 to PYCR3 and are widely distributed from mitochondria to cytoplasmic regions in various types of cells. While it has been known that PYCR2, together with PYCR1, plays a key role in synthesizing proline in mitochondria, PYCR3 (also called PYCRL) is also able to catalyze the final step in the biosynthesis of proline, converting pyrroline-5-carboxylate to proline in the cytoplasmic regions [15,16]. It remains to be determined why mutations of PYCR2 proteins are only associated with severe developing abnormalities throughout the whole brain, especially with abnormalities in oligodendroglial cells.

Studies on knockout mice of PYCR1 and PYCR2 show that PYCR1 and PYCR2 are redundant to synthesize proline [27]. PYCR1 and PYCR2 double knockout mice appear unhealthy but can survive [27,28], illustrating that the minimum amount of proline necessary for survival is supplied by PYCR3. It may thus be predicted that HLD10-associated PYCR2 mutated proteins display not only loss-of-function mutations for proline synthesis, but also somewhat toxic-gain-of-function mutations for other known and unknown intracellular functions. The recorded human PYCR2 3D structures (PBD No. 6LHM) show that mutated amino acids R119 and R251 are predicted to be positioned between the helical and sheet structures at the respective N- and C-terminal regions (Figure S18). It is conceivable that the mutations can not only inhibit the enzymatic activities, but also break to change the interactive abilities themselves between helical and sheet structures. It is known that PYCR2 increases the levels of glycine in brains through upregulation of serine hydroxymethyltransferase 2 (SHMT2), which is involved in catalyzing the forward and reverse biosynthetic reactions of serine and tetrahydrofolate to glycine and 5,10-methylene tetrahydrofolate as a major source of glycine and a minor source of serine [29]. Since PYCR2 indirectly has the abilities of activating the signaling pathway containing phosphatidylinositol-3 kinase (PI3K), Akt kinase, and mechanistic target of rapamycin (mTOR) [30], PYCR2 may be involved in the regulation of the relationship of intracellular energy balances with expression of some amino acid catabolizing- or metabolizing-related genes involving *shmt2*. It is conceivable that HLD10-associated PYCR2 mutations may also have inhibitory effects on the expression of amino acid catabolizing- or metabolizing-related genes to disturb cellular amino acid homeostasis. Despite this, it remains unknown how mutations in PYCR2 have specific effects on oligodendroglial cells.

We have found that cells expressing mutated PYCR2 proteins aggregate and form large size mitochondria. In addition, mitochondrial activities were decreased following expression of PYCR2 mutant proteins. One of the reasons for decreased mitochondrial activities may be that the mitochondrial size is too large to produce energy effectively. Alternatively, the nature of the connection of the intermembrane spaces themselves could be abnormal, inhibiting sequential respiratory molecular reactions. In either case, it is unlikely that such large size mitochondria are normally functional, leading to undifferentiated phenotypes. In contrast, cells with normal size mitochondria undergo differentiation. In the future, studies using patient cell and tissue samples will allow us to clarify the detailed relationships among mitochondrial sizes, mitochondrial activities, oligodendroglial cell undifferentiated phenotypes, and hypomyelinating phenotypes at the molecular, cellular, and tissue levels.

HLD4 is associated with the Asp29-to-Gly (D29G) mutation of mitochondrial heat shock 60-kDa protein 1 (HSPD1) [31]. This mutation causes short length mitochondrial morphologies, since the fission cycle is increased whereas the fusion cycle is decreased [32]. It also decreases the mitochondrial membrane potential [32]. Additionally, cells with small size mitochondria (expressing D29G mutated proteins) fail to exhibit differentiated phenotypes [32]. It is of interest that the cells with either small or large mitochondria observed in this study are associated with hypomyelinating disease phenotypes. It will be important to determine whether mitochondrial sizes or activities, or both are actually associated with oligodendroglial cell undifferentiated phenotypes and hypomyelinating ones.

Mutation of mitochondrial arginyl-tRNA synthetase 2 (RARS2) is known to be responsible for pontocerebellar hypoplasia type 6 (PCH6). PCH6 exhibits delayed myelinating phenotypes [33,34]. Similarly, mutation of mitochondrial prolyl-tRNA synthetase 2 (PARS2), which is associated with neurological diseases such as Alpers syndrome and some infantile-onset neurodegenerative disorders, is also accompanied by delayed myelinating phenotypes [35,36]. It is unknown if these myelin sheath–related diseases associated with mitochondrial residential proteins exhibit mitochondrial morphological abnormalities and decreased activities in mitochondria. Investigating the commonalities in the mitochondrial residential protein-associated diseases, including HLD4 and HLD10, may shed light on which types of molecular mechanisms underly the myelin sheath-related diseases.

Herein we have demonstrated that HLD10-associated PYCR2 mutant proteins aggregate and lead to the formation of large size mitochondria, resulting in a decrease in mitochondrial activities. Cells expressing PYCR2 mutant proteins fail to undergo oligodendroglial cell morphological differentiation, whereas cells expressing wild type proteins normally undergo differentiation. Further studies will allow us to clarify whether treatment of cells with chemically synthesized cell-permeable proline will simply recover aberrant cellular phenotypes, since HLD10 is presently thought to be associated with loss-of-function [9,10,11,12,13,14,15,16]. If treatment with cell-permeable proline is not enough to recover cellular phenotypes, HLD10-associated PYCR2 mutations may contain gain-of-function, likely causing the expression of genes controlling protein and mitochondrial morphological stabilities as well as metabolism and catabolism. Additional studies will promote our understanding not only of the detailed mechanism by which HLD10-associated PYCR2 mutations form large size mitochondria, but also by which their mutations are related to inhibiting morphological differentiation in cells and possibly in mice (such as transgenic mutations). Studies related to these disease biological fields might lead to the development of drug target-specific medicine candidates for HLD10 and probable HLD4 as well as other mitochondrial leukodystrophies.

## Data Availability

Not applicable.

## Supplementary Materials

The following supporting information can be downloaded at: https://www.mdpi.com/article/10.3390/neurolint14040085/s1, Figure S1: The wild type PYCR2 proteins do not exhibit aggregation in cells.; Figure S2. The R119C proteins of PYCR2 exhibit aggregation in cells.; Figure S3: The R252C proteins of PYCR2 exhibit aggregation in cells.; Figure S4: The wild type (WT) proteins of PYCR2 are not localized in the ER.; Figure S5: The R119C proteins of PYCR2 are not localized in the ER.; Figure S6: The R251C proteins of PYCR2 are not localized in the ER.Figure S7: The wild type (WT) proteins of PYCR2 are not localized in the Golgi body.; Figure S8: The R119C proteins of PYCR2 are not localized in the Golgi body.; Figure S9: The R251C proteins of PYCR2 are not localized in the Golgi body.; Figure S10: The wild type (WT) proteins of PYCR2 are not localized in the lysosome.; Figure S11: The R119C proteins of PYCR2 are not localized in the lysosome. Figure S12: The R251C proteins of PYCR2 are not localized in the lysosome.; Figure S13: The wild type (WT) proteins of PYCR2 are localized in the mitochondrion.; Figure S14: The R119C proteins of PYCR2 are localized in the mitochondrial large size punctate structures.; Figure S15: The R251C proteins of PYCR2 are localized in the mitochondrial large size punctate structures.; Figure S16: The R119C or R251C proteins increase mitochondrial fusion.; Figure S17: Cells expressing the R119C or R251C proteins fail to exhibit oligodendroglial cell morphological differentiation.; Figure S18: Predicted 3D structures of PYCR2 and the mutated positions.; and Figure S19: Full-size gel images in the immunoblots of Figure 9.
