# Supplementary material for: Hypomyelinating Leukodystrophy 10 (HLD10)-Associated Mutations of PYCR2 Form Large Size Mitochondria, Inhibiting Oligodendroglial Cell Morphological Differentiation
Source: Neurol Int. 2022 Dec 16;14(4):1062–80. doi: 10.3390/neurolint14040085 (PMC9787162; doi:10.3390/neurolint14040085)
Supplement: Supplementary file 1 [file neurolint-14-00085-s001.zip › neurolint-1970436-supplementary.pdf]

Supplementary figure legends

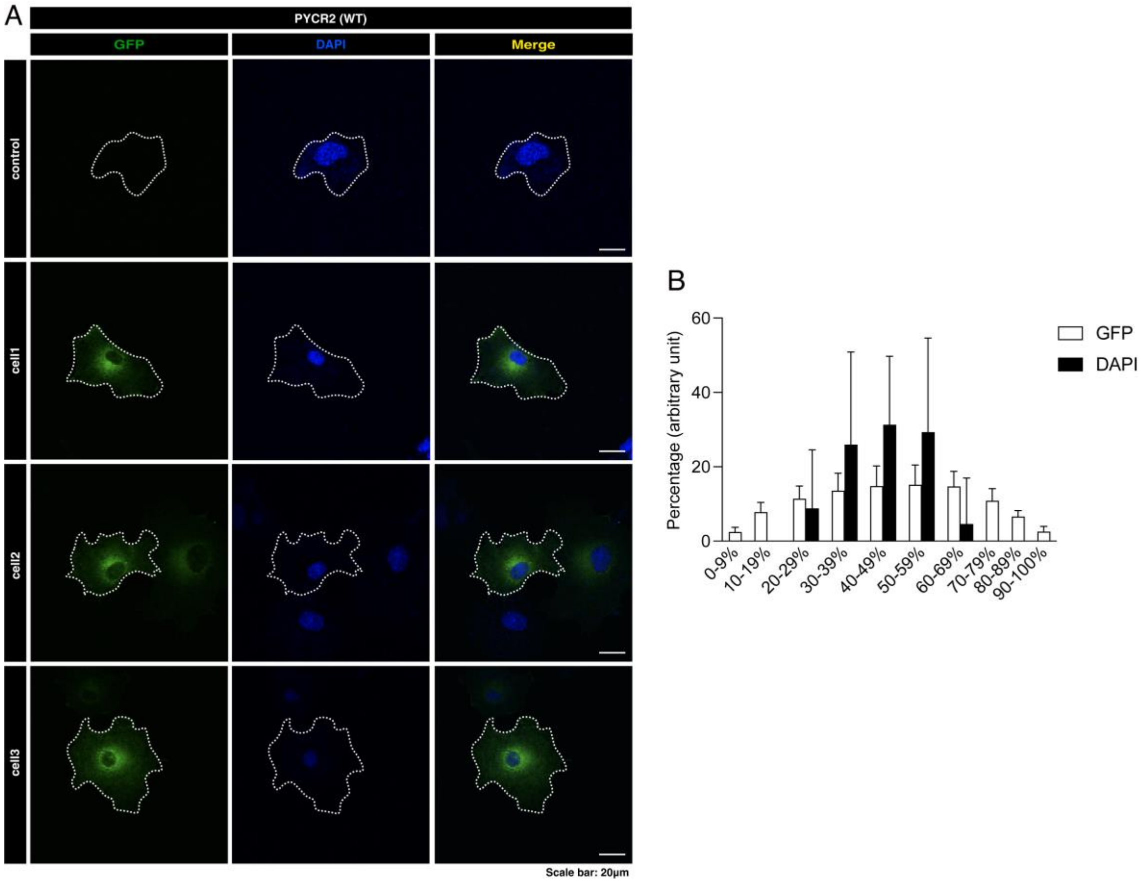

Figure S1. The wild type (WT) proteins of PYCR2 are not localized in the ER. (A, B) COS-7 cells were transfected with the plasmid encoding the wild type PYCR2 (green) and stained with an anti-KDEL antibody (red). The approximate outline of the cell is surrounded by white lines. Scan plots along white dotted lines in the direction of the arrows were performed. Graphs showing fluorescent intensities (F.I., arbitrary unit) were depicted in the bottom right panels.

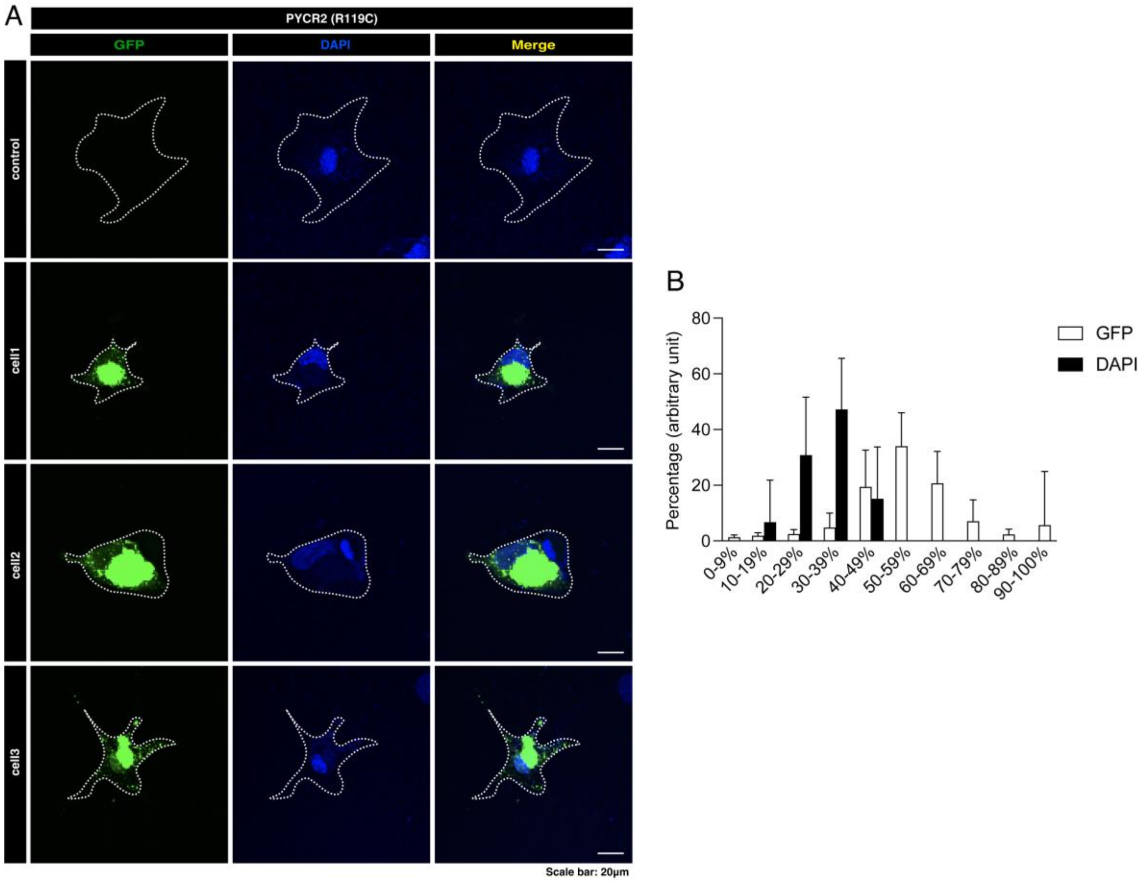

Figure S2. The R119C proteins of PYCR2 are not localized in the ER. (A, B) COS-7 cells were transfected with the plasmid encoding the PYCR2 R119C (green) and stained with an anti-KDEL antibody (red). The approximate outline of the cell is surrounded by white lines. Scan plots along white dotted lines in the direction of the arrows were performed. Graphs showing fluorescent intensities (F.I., arbitrary unit) were depicted in the bottom right panels.

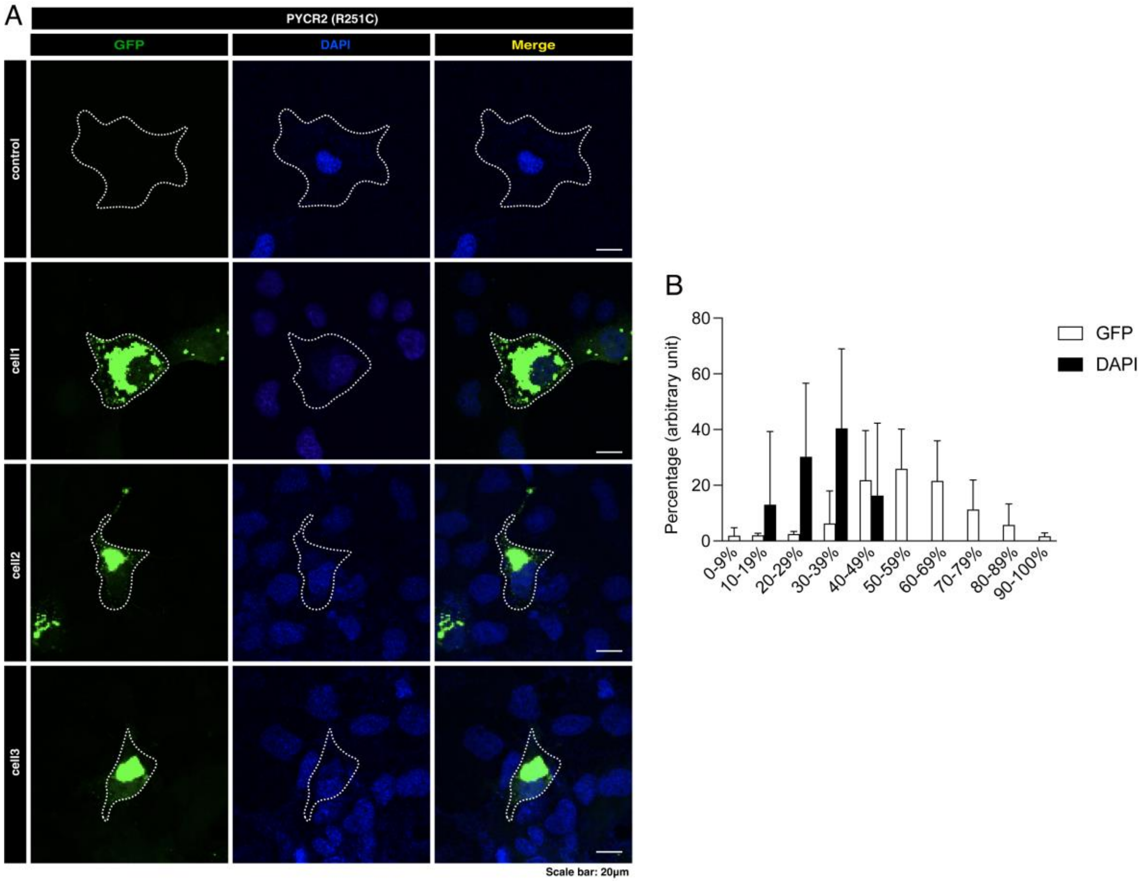

Figure S3. The R251C proteins of PYCR2 are not localized in the ER. (A, B) COS-7 cells were transfected with the plasmid encoding the PYCR2 R251C (green) and stained with an anti-KDEL antibody (red). The approximate outline of the cell is surrounded by white lines. Scan plots along white dotted lines in the direction of the arrows were performed. Graphs showing fluorescent intensities (F.I., arbitrary unit) were depicted in the bottom right panels.

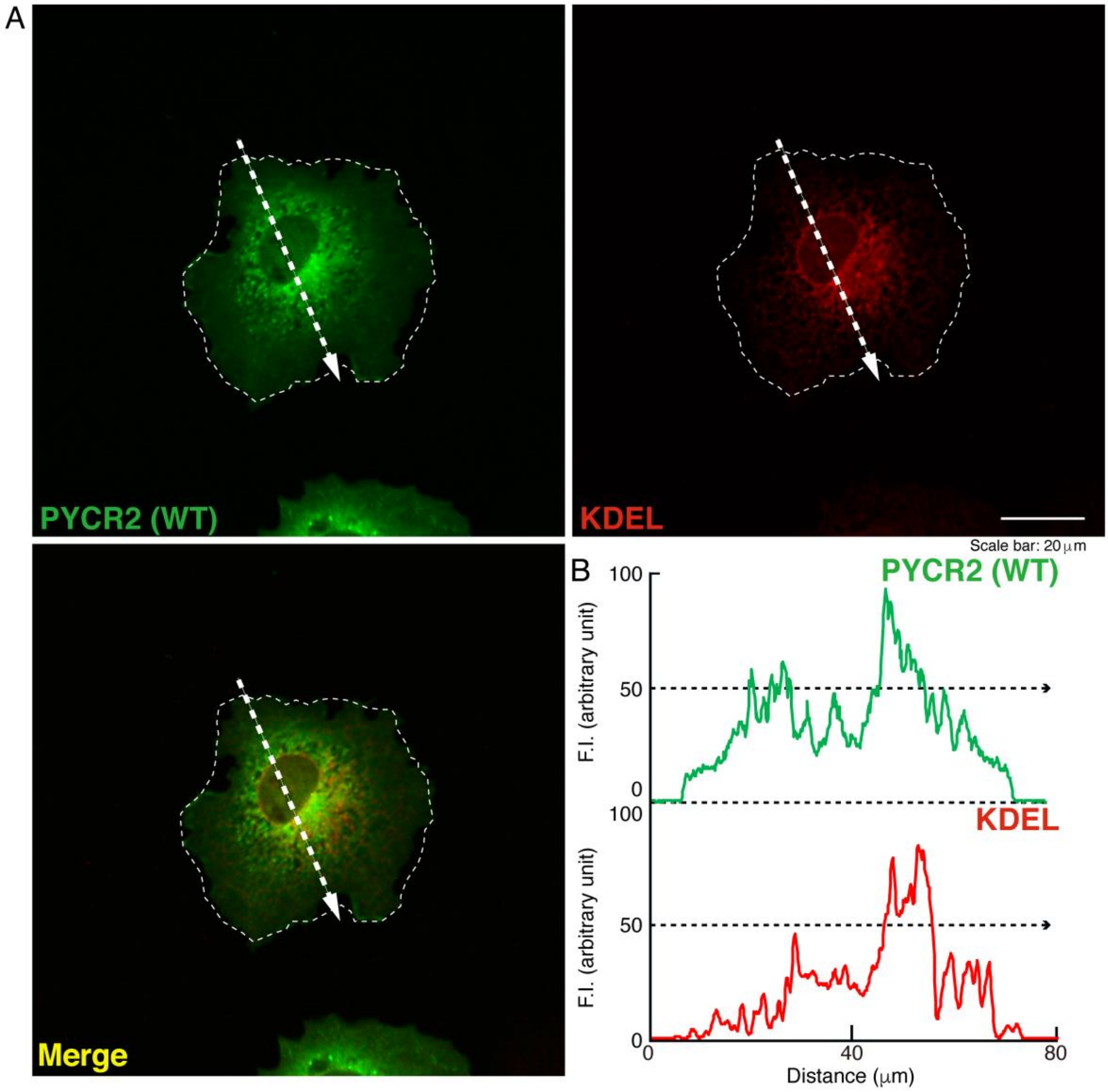

Figure S4. The wild type (WT) proteins of PYCR2 are not localized in the Golgi body. (A, B) COS-7 cells were transfected with the plasmid encoding the wild type PYCR2 (green) and stained with an anti-GM130 antibody (red). The approximate outline of the cell is surrounded by white lines. Scan plots along white dotted lines in the direction of the arrows were performed. Graphs showing fluorescent intensities (F.I., arbitrary unit) were depicted in the bottom right panels.

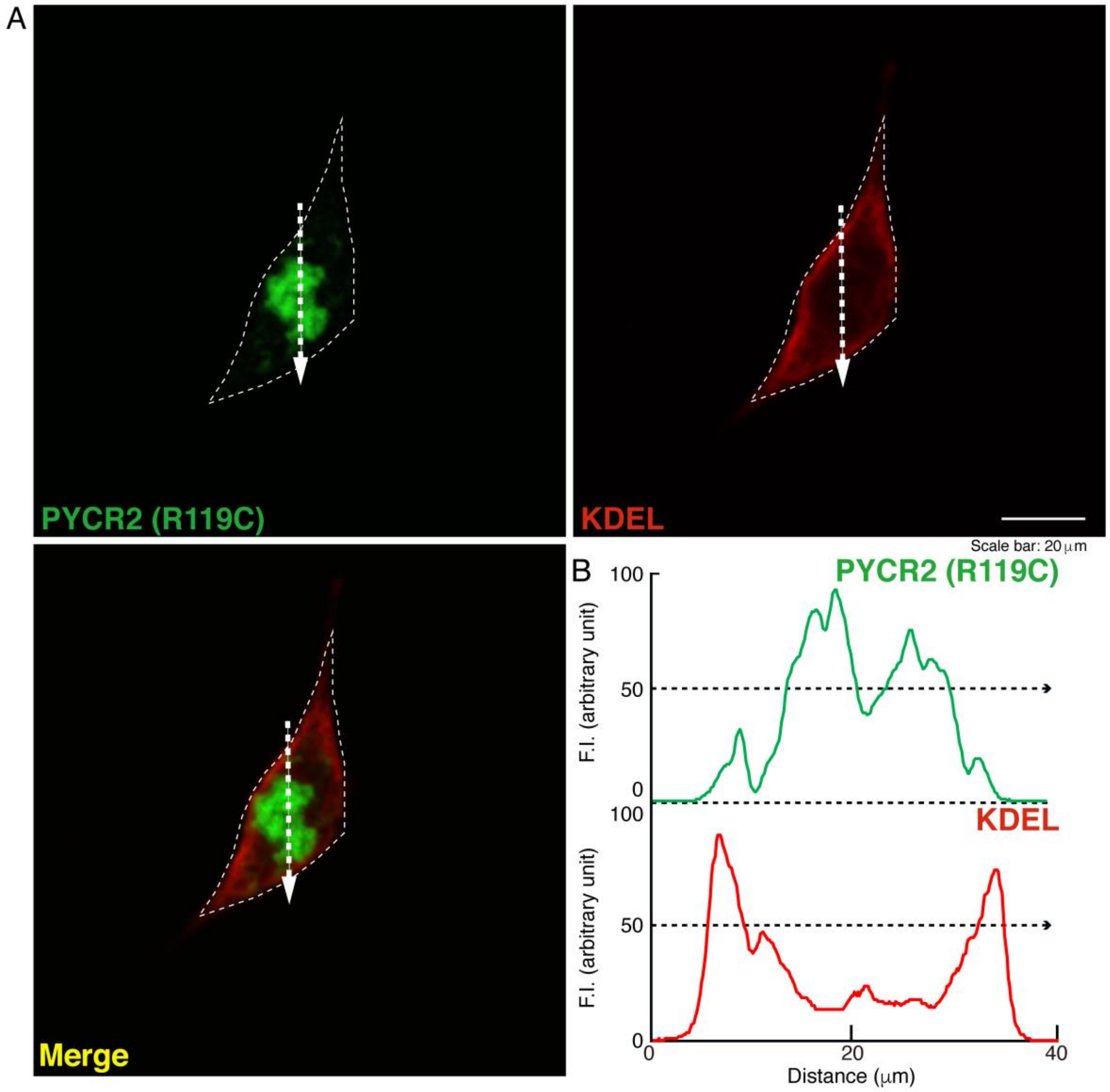

Figure S5. The R119C proteins of PYCR2 are not localized in the Golgi body. (A, B) COS-7 cells were transfected with the plasmid encoding the PYCR2 R119C (green) and stained with an anti-GM130 antibody (red). The approximate outline of the cell is surrounded by white lines. Scan plots along white dotted lines in the direction of the arrows were performed. Graphs showing fluorescent intensities (F.I., arbitrary unit) were depicted in the bottom right panels.

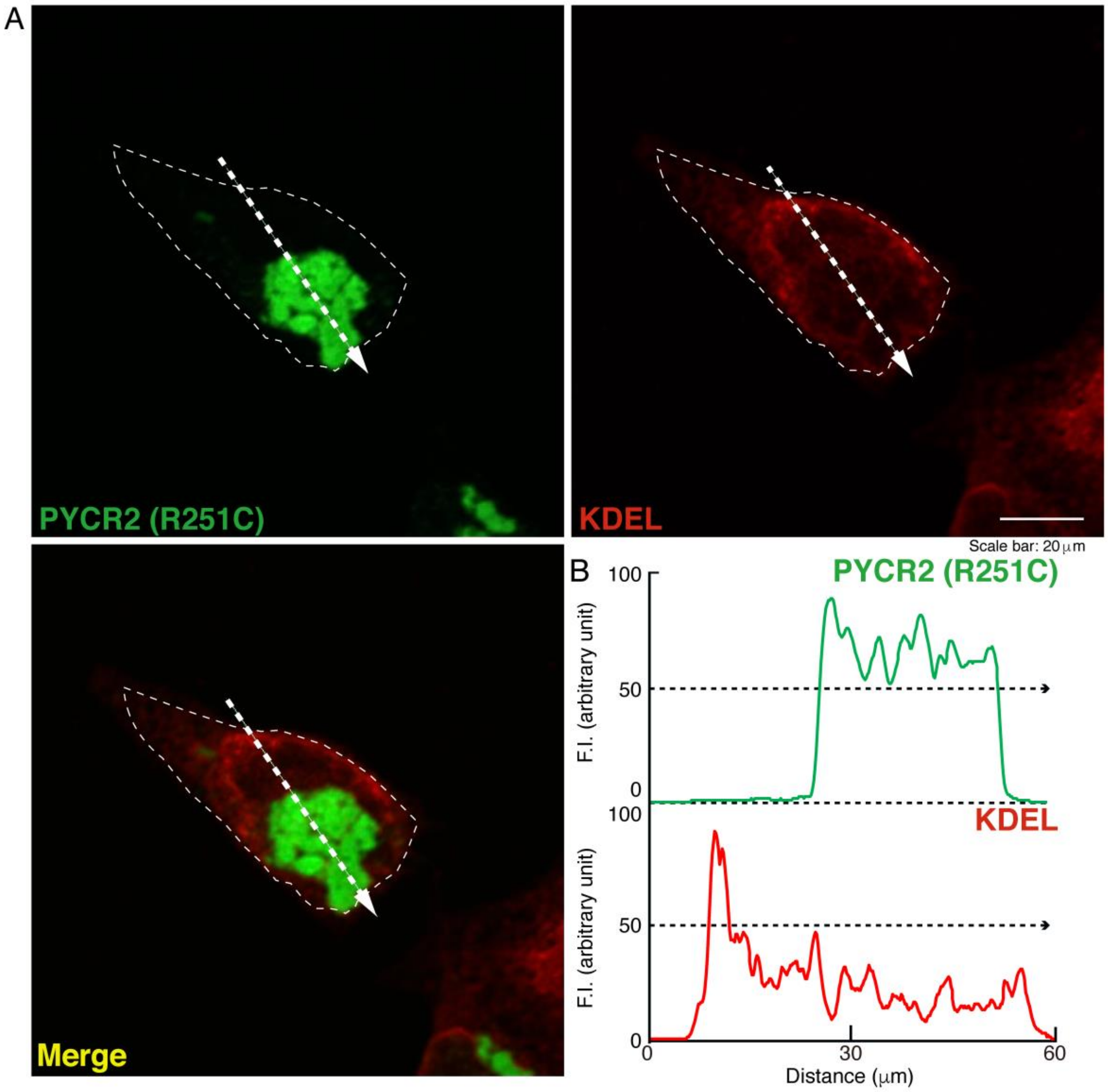

Figure S6. The R251C proteins of PYCR2 are not localized in the Golgi body. (A, B) COS-7 cells were transfected with the plasmid encoding the PYCR2 R251C (green) and stained with an anti-GM130 antibody (red). The approximate outline of the cell is surrounded by white lines. Scan plots along white dotted lines in the direction of the arrows were performed. Graphs showing fluorescent intensities (F.I., arbitrary unit) were depicted in the bottom right panels.

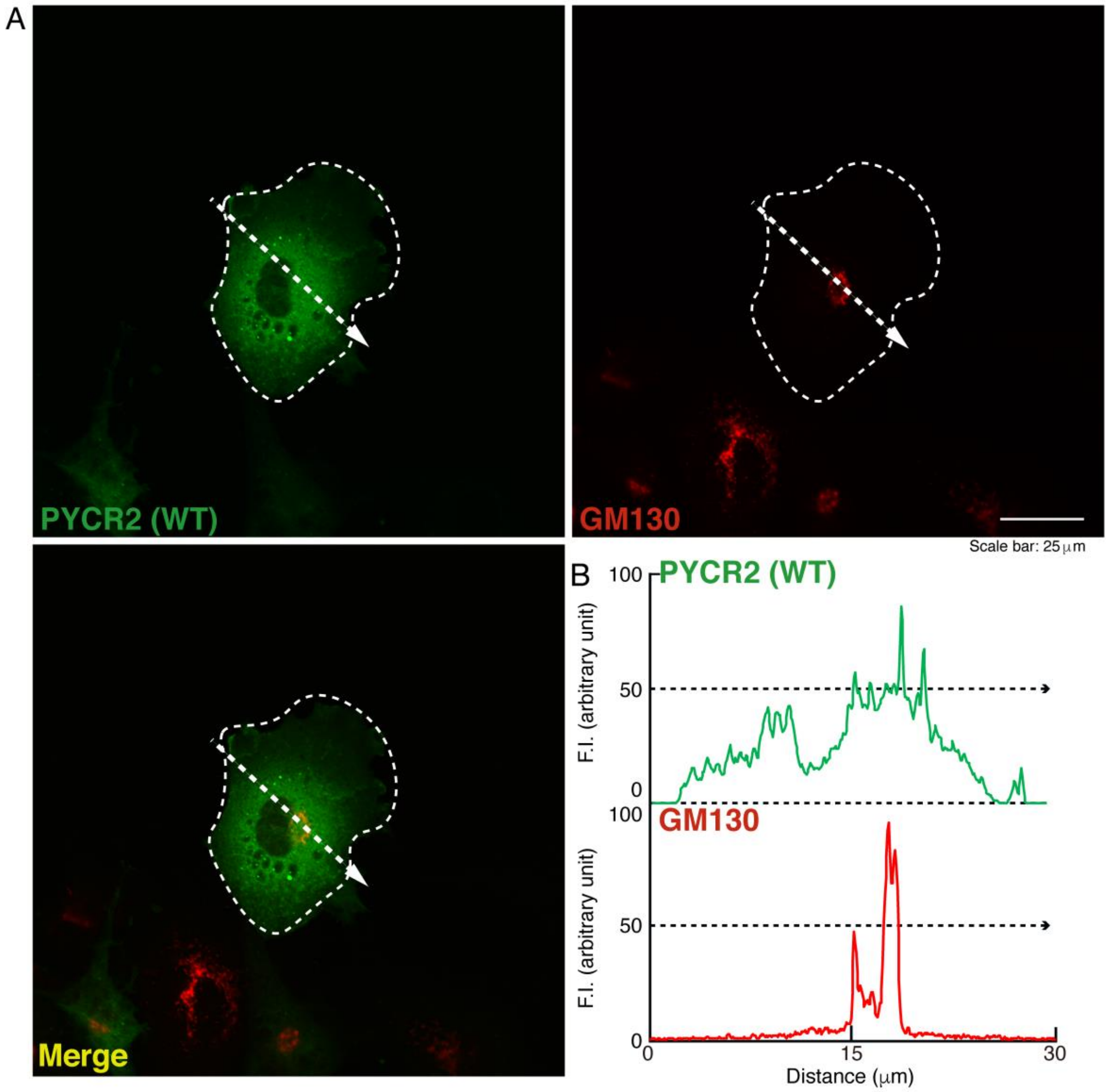

Figure S7. The wild type (WT) proteins of PYCR2 are not localized in the lysosome. (A, B) COS-7 cells were transfected with the plasmid encoding the wild type PYCR2 (green) and stained with an anti-LAMP1 antibody (red). The approximate outline of the cell is surrounded by white lines. Scan plots along white dotted lines in the direction of the arrows were performed. Graphs showing fluorescent intensities (F.I., arbitrary unit) were depicted in the bottom right panels.

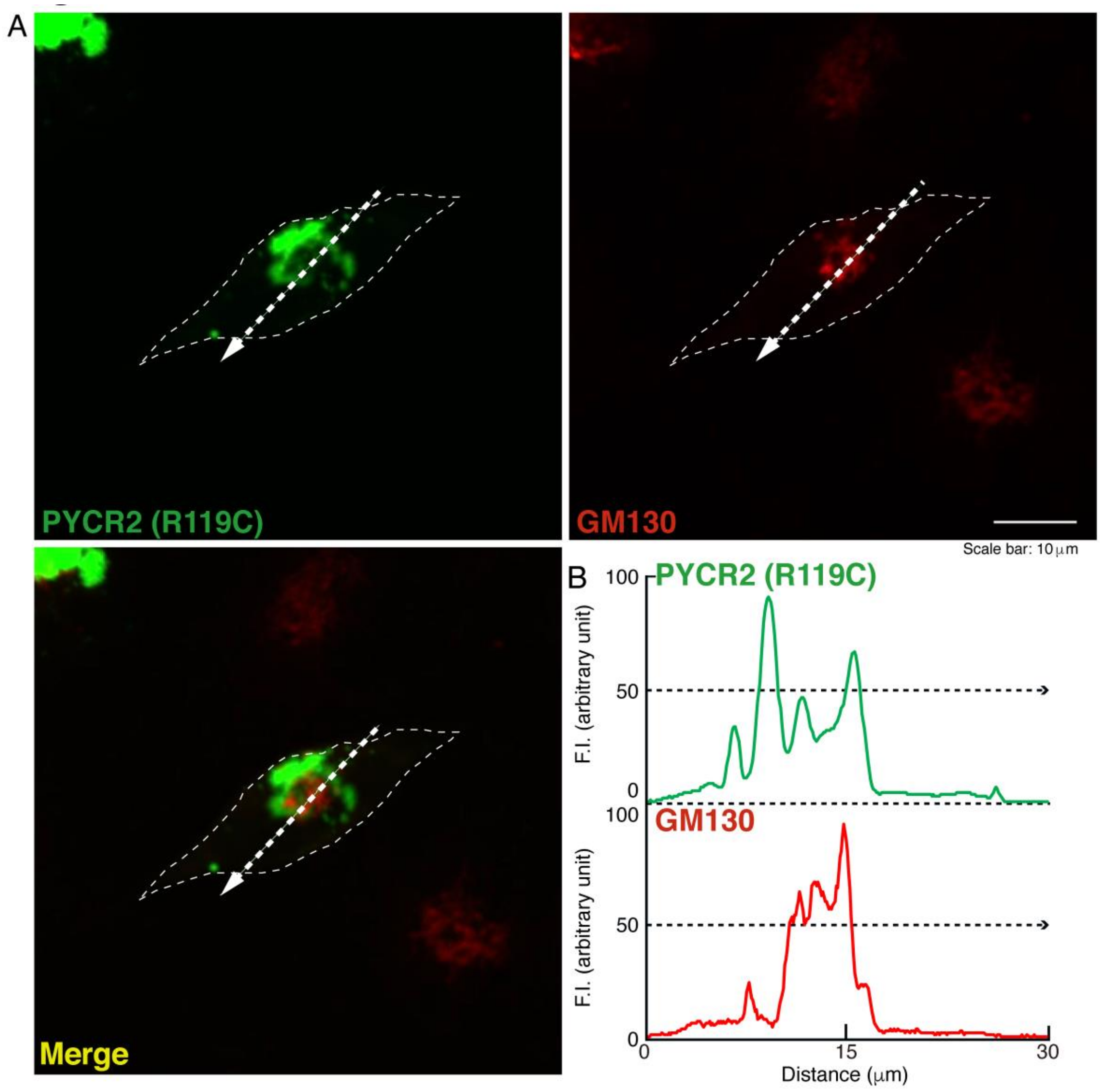

Figure S8. The R119C proteins of PYCR2 are not localized in the lysosome. (A, B) COS-7 cells were transfected with the plasmid encoding the PYCR2 R119C (green) and stained with an anti-LAMP1 antibody (red). The approximate outline of the cell is surrounded by white lines. Scan plots along white dotted lines in the direction of the arrows were performed. Graphs showing fluorescent intensities (F.I., arbitrary unit) were depicted in the bottom right panels.

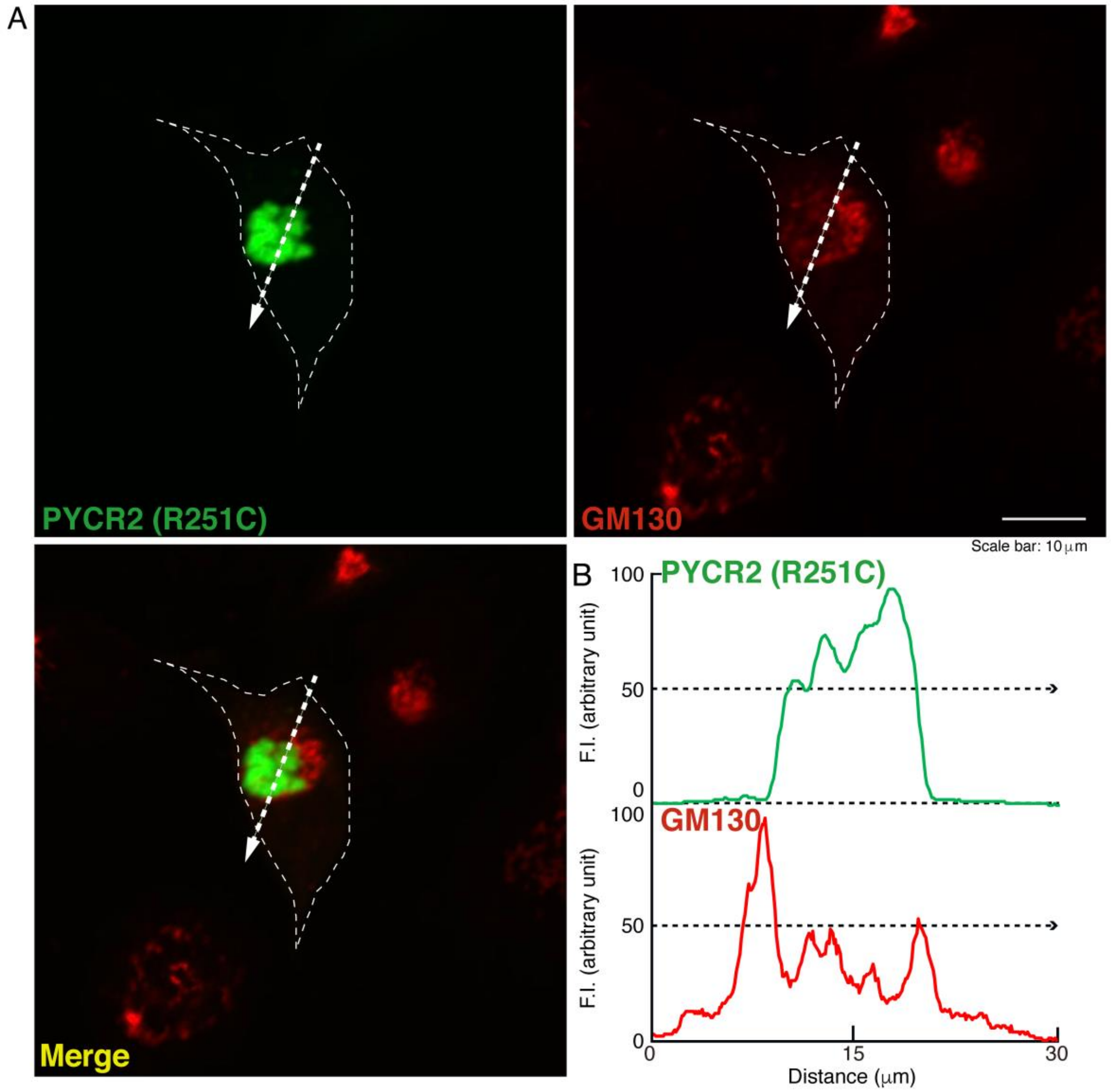

Figure S9. The R251C proteins of PYCR2 are not localized in the lysosome. (A, B) COS-7 cells were transfected with the plasmid encoding the PYCR2 R251C (green) and stained with an anti-LAMP1 antibody (red). The approximate outline of the cell is surrounded by white lines. Scan plots along white dotted lines in the direction of the arrows were performed. Graphs showing fluorescent intensities (F.I., arbitrary unit) were depicted in the bottom right panels.

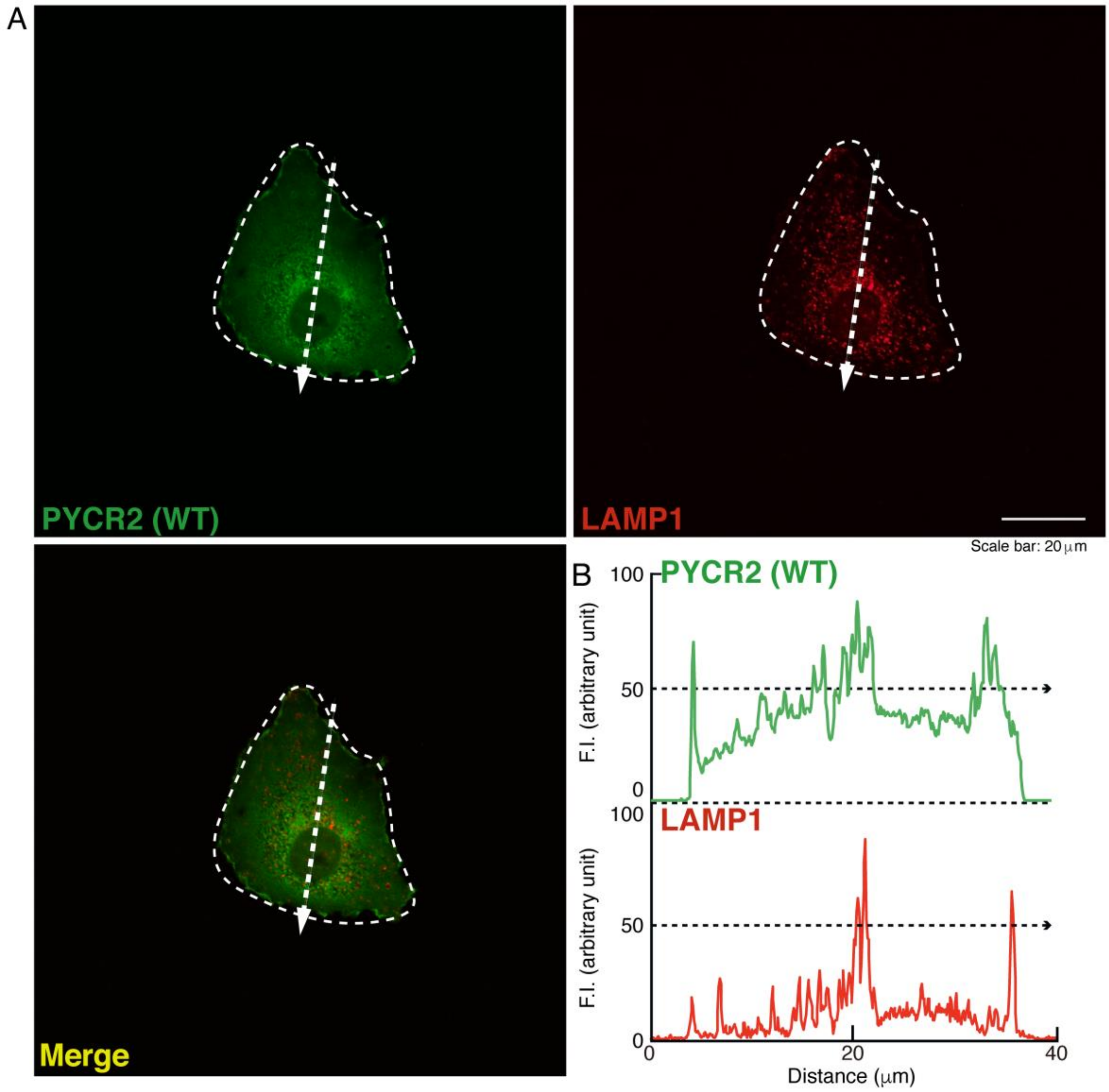

Figure S10. The wild type (WT) proteins of PYCR2 are localized in the mitochondrion. (A, B) FBD-102b cells were transfected with the plasmid encoding the wild type PYCR2 (green) and stained with an anti-HSPD1 antibody (red). The approximate outline of the cell is surrounded by white lines. Scan plots along white dotted lines in the direction of the arrows were performed. Graphs showing fluorescent intensities (F.I., arbitrary unit) were depicted in the bottom right panels.

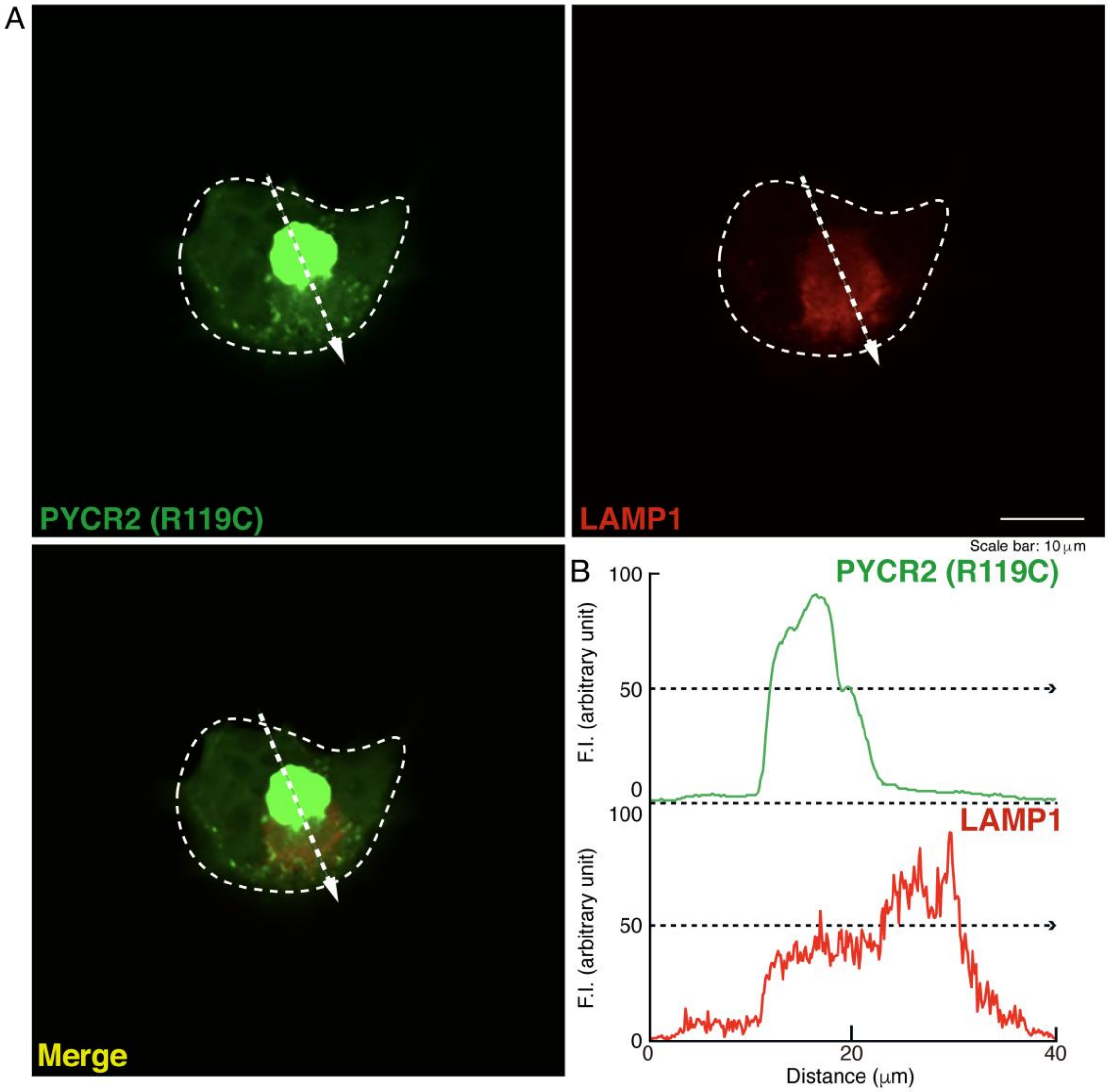

Figure S11. The R119C proteins of PYCR2 are localized in the mitochondrial large size punctate structures. (A, B) FBD-102b cells were transfected with the plasmid encoding the PYCR2 R119C (green) and stained with an anti-HSPD1 antibody (red). The approximate outline of the cell is surrounded by white lines. Scan plots along white dotted lines in the direction of the arrows were performed. Graphs showing fluorescent intensities (F.I., arbitrary unit) were depicted in the bottom right panels.

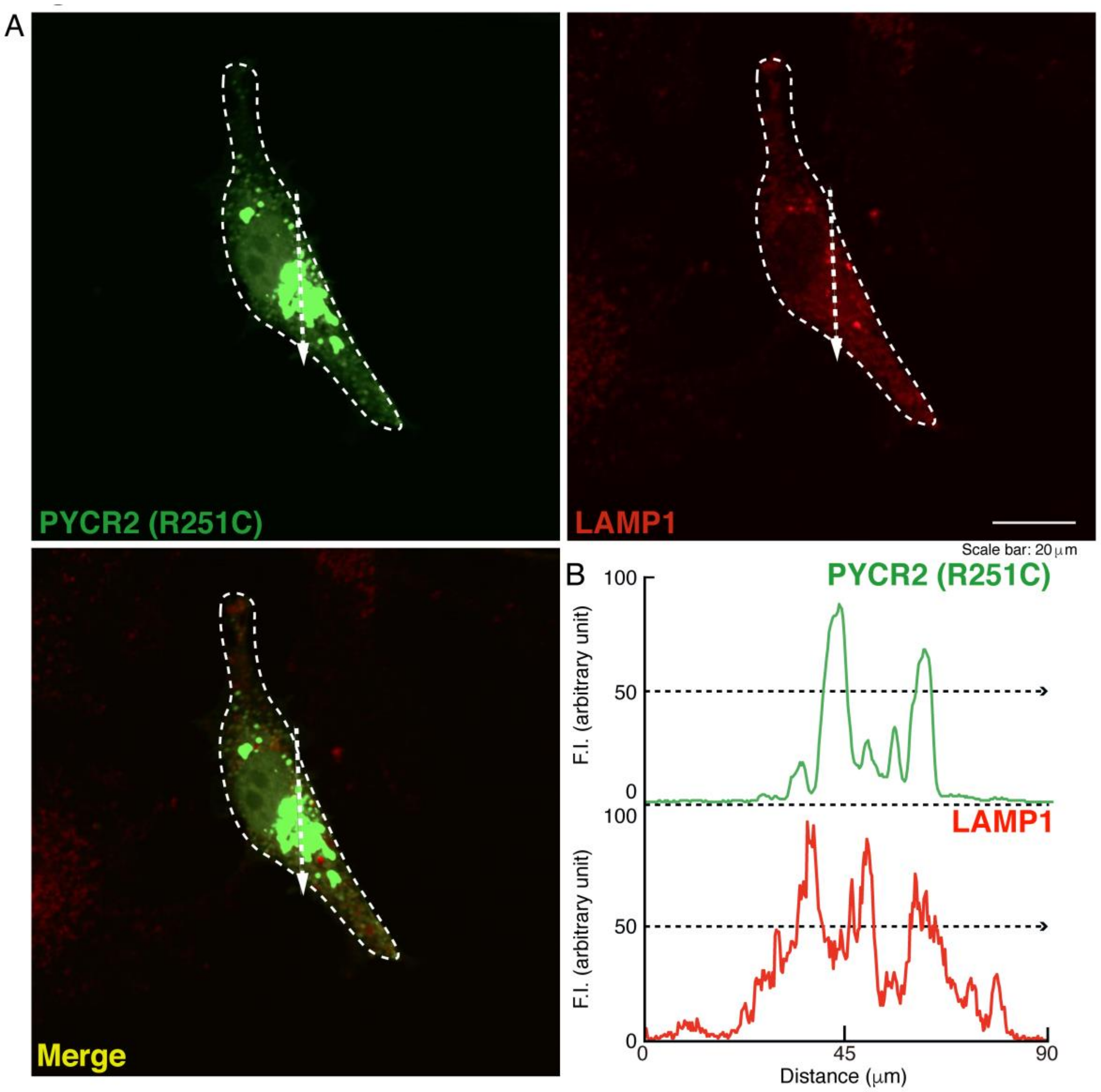

Figure S12. The R251C proteins of PYCR2 are localized in the mitochondrial large size punctate structures. (A, B) FBD-102b cells were transfected with the plasmid encoding the PYCR2 R251C (green) and stained with an anti-HSPD1 antibody (red). The approximate outline of the cell is surrounded by white lines. Scan plots along white dotted lines in the direction of the arrows were performed. Graphs showing fluorescent intensities (F.I., arbitrary unit) were depicted in the bottom right panels.

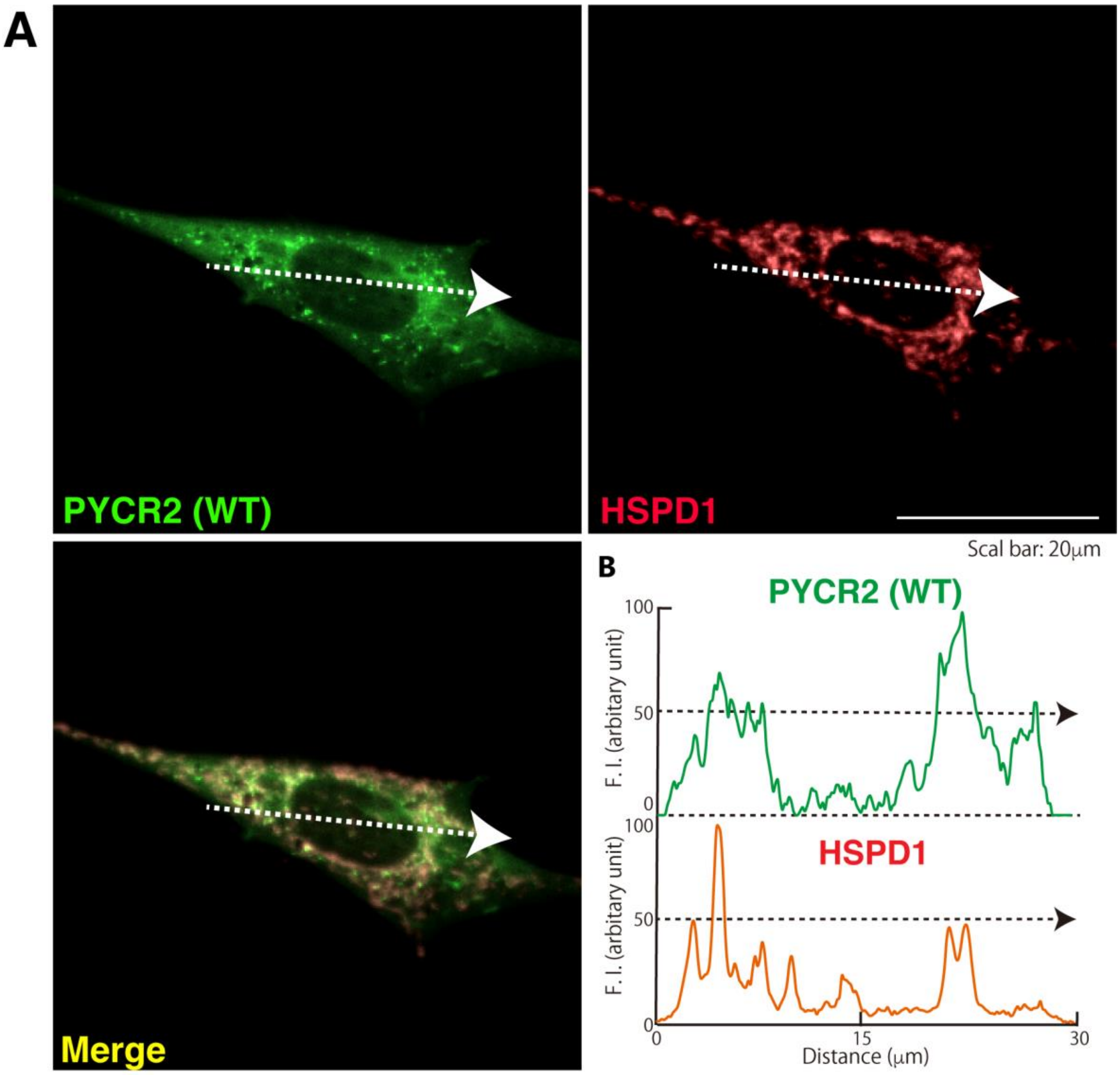

Figure S13. The mutated PYCR2 proteins decrease mitochondrial membrane potentials. (A, B) FBD-102b cells expressing wild type (WT), R119C, or R251C of PYCR2 (green) and treated with JC-1 dye (red). The approximate outline of the cell is surrounded by white lines. JC-1 was taken up by wild type PYCR2-expressing mitochondria that maintained normal membrane potential. Pixel ratios of red fluorescence per green fluorescence were depicted to be statistically compared.

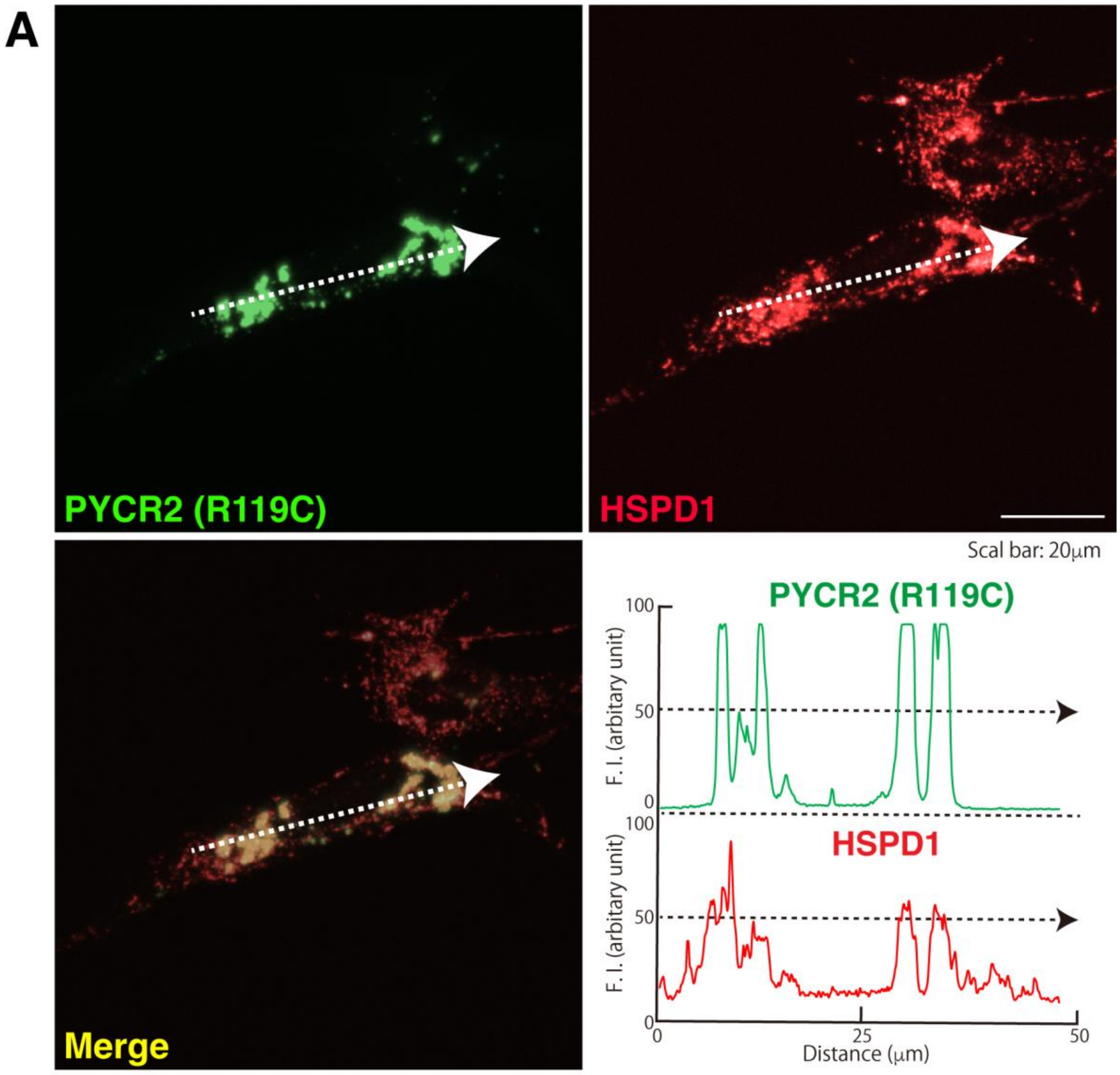

Figure S14. Predicted 3D structures of PYCR2 and the mutated positions. (A, B) Predicted dimeric 3D structures of PYCR2 and mutated amino acid positions were depicted from different directions.

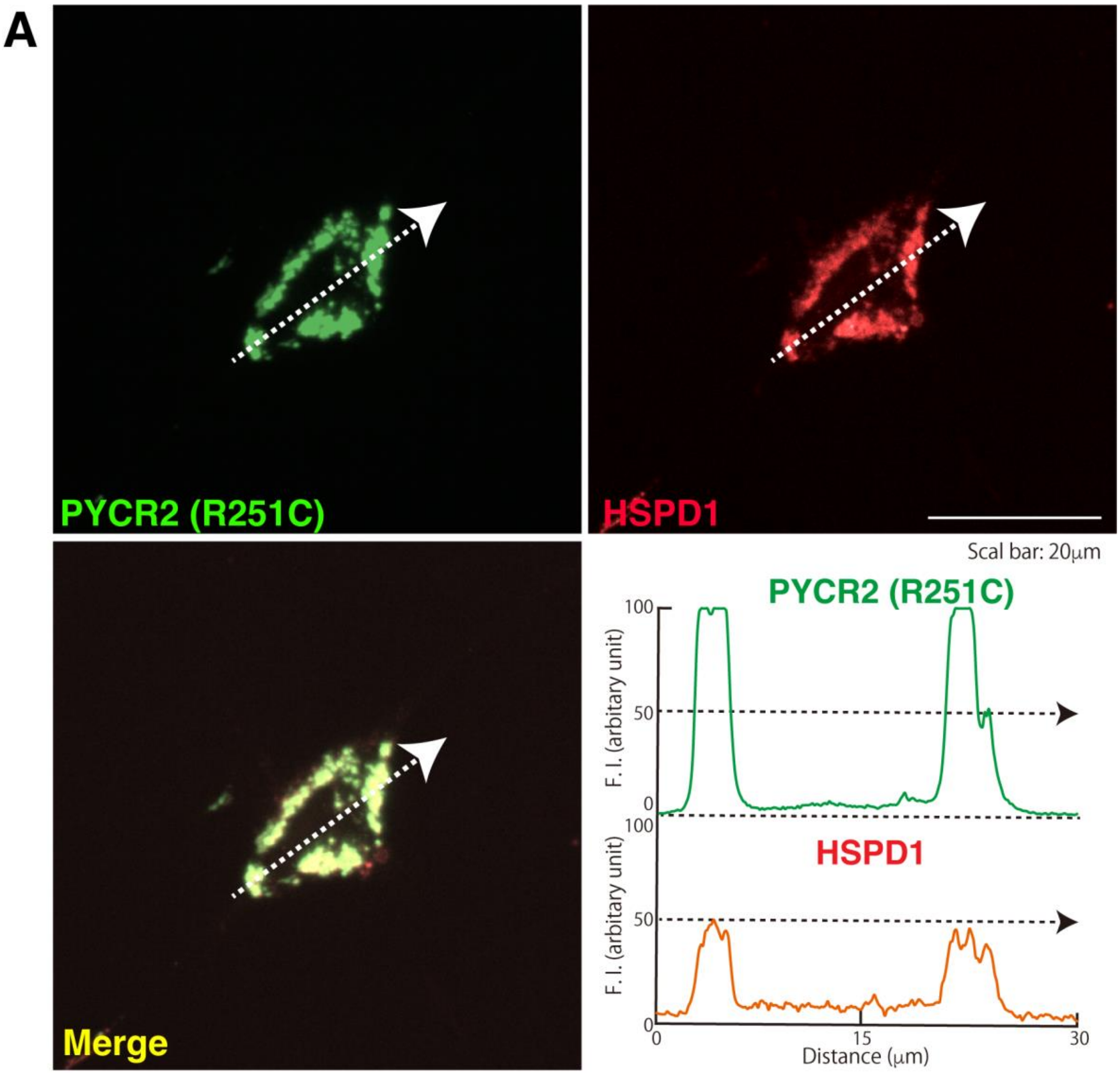

Figure S15. Full size gel images in immunoblots of the Figure 8

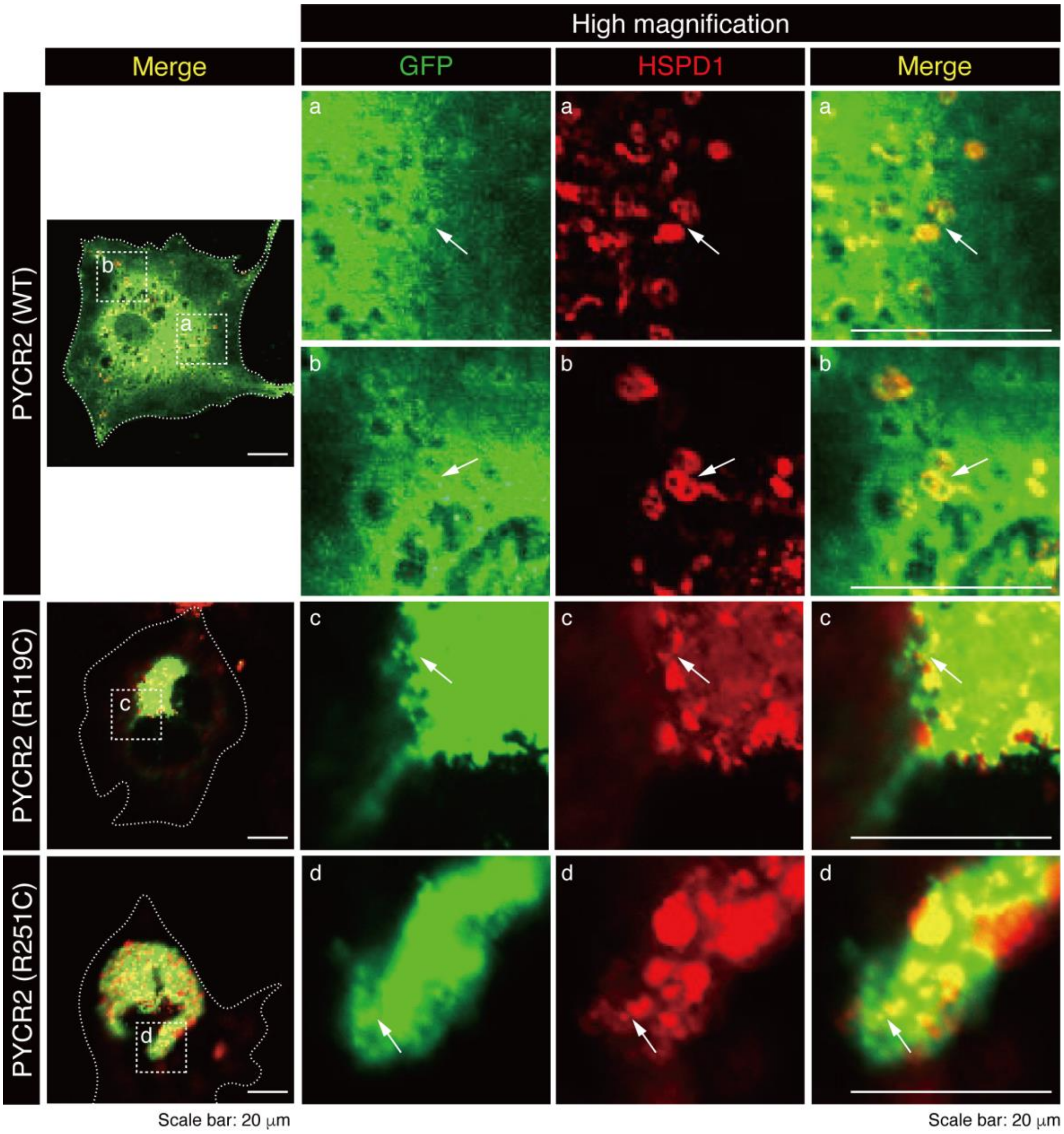

Figure S16: The R119C or R251C proteins increase mitochondrial fusion.

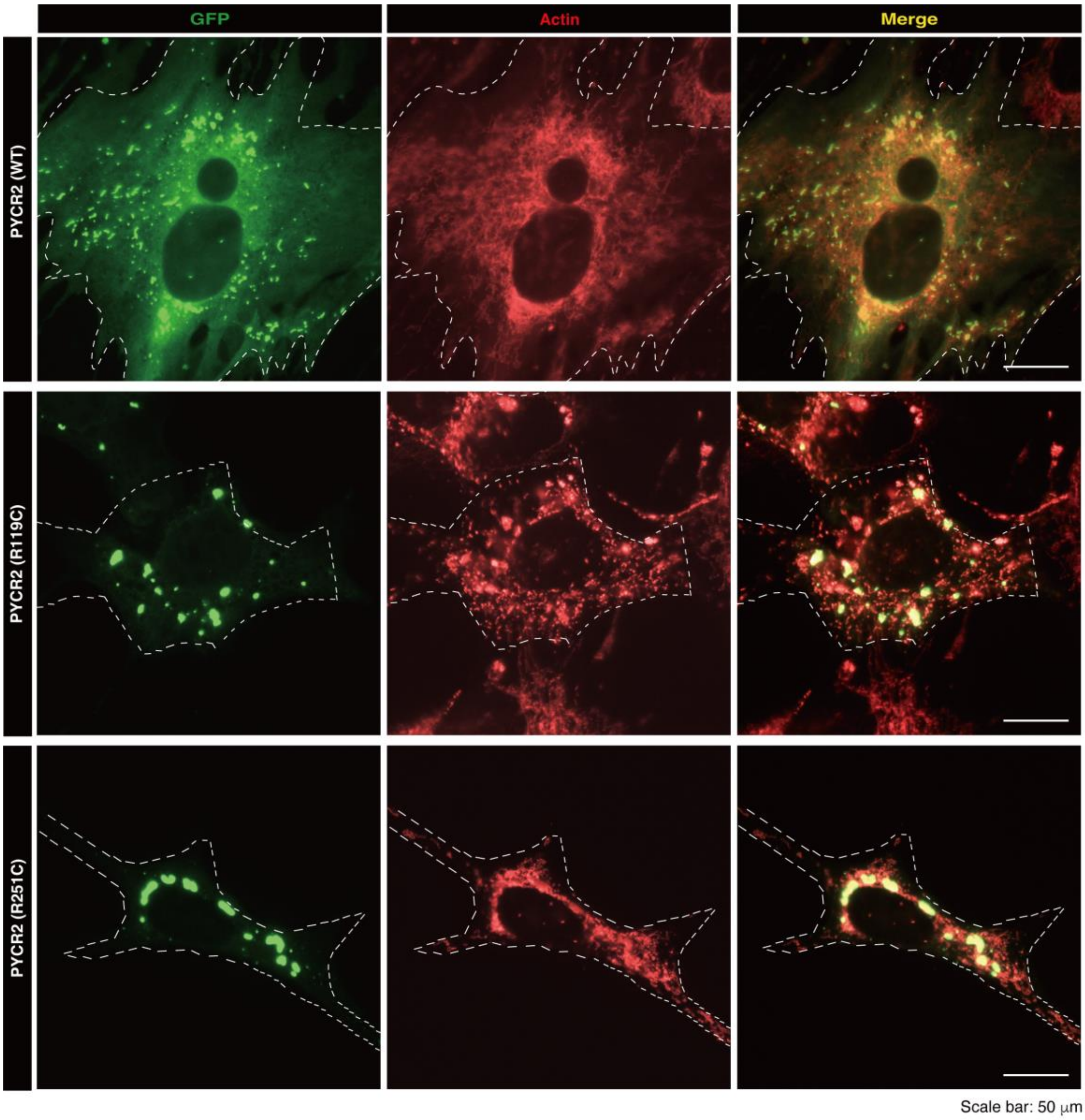

Figure S17: Cells expressing the R119C or R251C proteins fail to exhibit oligodendroglial cell morphological differentiation.

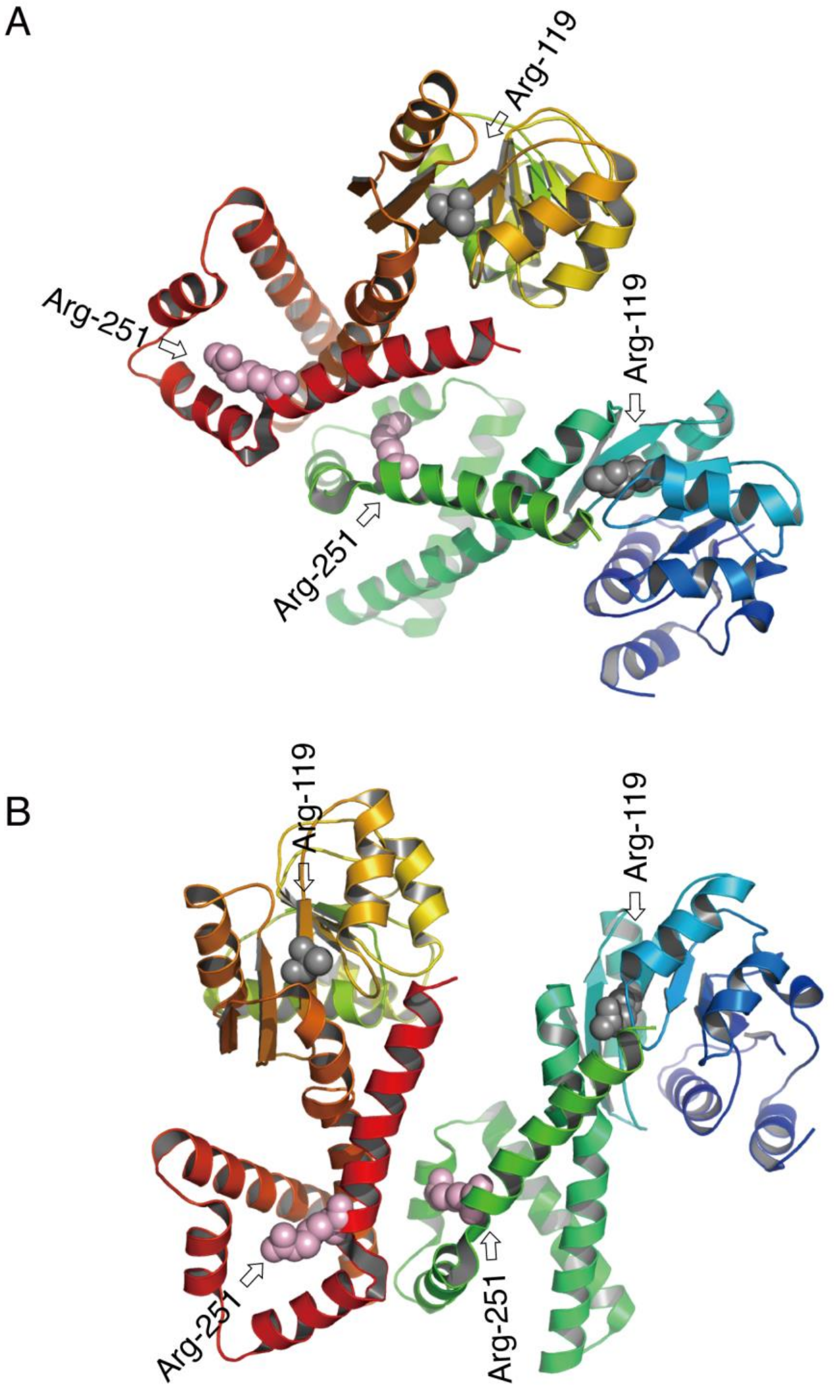

Figure S18: Predicted 3D structures of PYCR2 and the mutated positions.

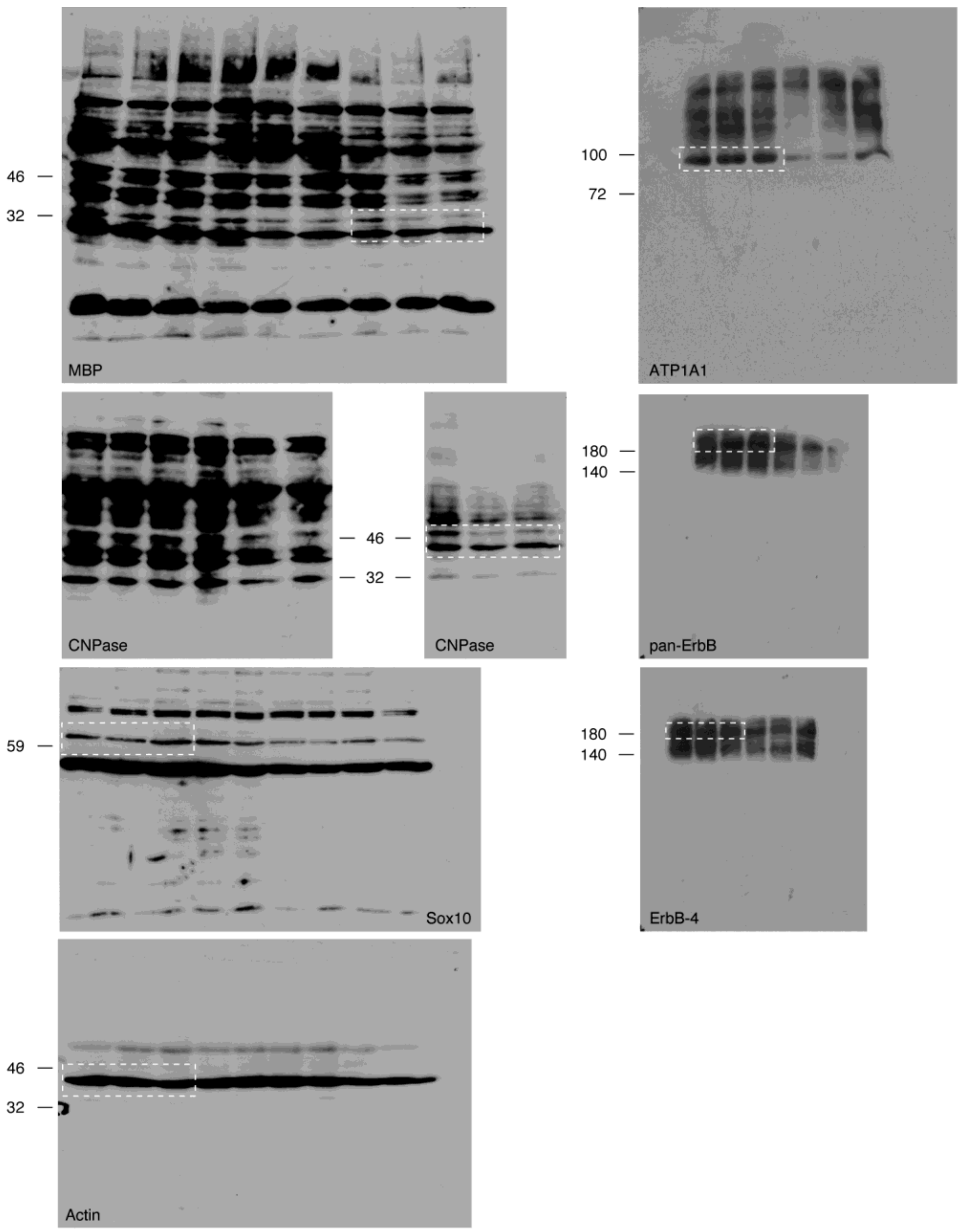

Figure S19: Full-size gel images in the immunoblots of Figure 9.
